# Unusual Cytochrome *c*552 from *Thioalkalivibrio paradoxus*: Solution NMR Structure and Interaction with Thiocyanate Dehydrogenase

**DOI:** 10.3390/ijms23179969

**Published:** 2022-09-01

**Authors:** Vladimir V. Britikov, Eduard V. Bocharov, Elena V. Britikova, Natalia I. Dergousova, Olga G. Kulikova, Anastasia Y. Solovieva, Nikolai S. Shipkov, Larisa A. Varfolomeeva, Tamara V. Tikhonova, Vladimir I. Timofeev, Eleonora V. Shtykova, Dmitry A. Altukhov, Sergey A. Usanov, Alexander S. Arseniev, Tatiana V. Rakitina, Vladimir O. Popov

**Affiliations:** 1 Institute of Bioorganic Chemistry, National Academy of Sciences of Belarus, 220141 Minsk, Belarus; 2 Shemyakin-Ovchinnikov Institute of Bioorganic Chemistry, Russian Academy of Sciences, Moscow 117997, Russia; 3 Moscow Institute of Physics and Technology, Dolgoprudny 141700, Russia; 4 Bach Institute of Biochemistry, Federal Research Center “Fundamentals of Biotechnology”, Russian Academy of Sciences, Moscow 119071, Russia; 5 Federal Scientific Research Center “Crystallography and Photonics”, Russian Academy of Sciences, Moscow 119333, Russia; 6 National Research Center “Kurchatov Institute”, Moscow 123182, Russia

**Keywords:** cytochrome *c*, thiocyanate dehydrogenase, electron transfer, intrinsically disordered protein, heme axial methionine fluxionality, protein complex modeling

## Abstract

The search of a putative physiological electron acceptor for thiocyanate dehydrogenase (TcDH) newly discovered in the thiocyanate-oxidizing bacteria *Thioalkalivibrio paradoxus* revealed an unusually large, single-heme cytochrome *c* (CytC552), which was co-purified with TcDH from the periplasm. Recombinant CytC552, produced in *Escherichia coli* as a mature protein without a signal peptide, has spectral properties similar to the endogenous protein and serves as an in vitro electron acceptor in the TcDH-catalyzed reaction. The CytC552 structure determined by NMR spectroscopy reveals significant differences compared to those of the typical class I bacterial cytochromes *c*: a high solvent accessible surface area for the heme group and so-called “intrinsically disordered” nature of the histidine-rich N- and C-terminal regions. Comparison of the signal splitting in the heteronuclear NMR spectra of oxidized, reduced, and TcDH-bound CytC552 reveals the heme axial methionine fluxionality. The TcDH binding site on the CytC552 surface was mapped using NMR chemical shift perturbations. Putative TcDH-CytC552 complexes were reconstructed by the information-driven docking approach and used for the analysis of effective electron transfer pathways. The best pathway includes the electron hopping through His528 and Tyr164 of TcDH, and His83 of CytC552 to the heme group in accordance with pH-dependence of TcDH activity with CytC552.

## 1. Introduction

Chemolithoautotrophic sulfur-oxidizing bacteria (SOB) are widely distributed in nature and play an important role in the metabolism of various sulfur compounds. The dominant group of chemolithotrophic SOB found in soda lakes around the world belongs to the genus *Thioalkalivibrio* (family Ectothiorhodospiraceae, class Gammaproteobacteria). They are obligate haloalkalophiles capable of growth at pH 10 and a salt concentration of 0.5–4 M Na^+^. It was shown that some strains of *Thioalkalivibrio* are able to grow on thiocyanate as the only source of nitrogen and electrons [1,2,3].

Thiocyanate (SCN^−^) is formed as a byproduct in several industrial processes, such as the coal and gold mining industries [4,5,6]. Although thiocyanate is not as toxic as cyanide, this stable compound is difficult to remove from the environment using chemical methods [7,8]. One of the approaches to the purification of industrial wastewater from thiocyanate is the use of SOB [6,9], including haloalkalophilic bacteria of the genus *Thioalkalivibrio* [1], which decompose thiocyanate producing sulfate and ammonium as terminal products. The first stage of thiocyanate decomposition can be catalyzed by either thiocyanate hydrolase or recently discovered thiocyanate dehydrogenase (TcDH, EC1.8.2.7) [3].

TcDH was isolated from the thiocyanate-oxidizing bacterium *T. paradoxus* ARh 1 and a novel molecular mechanism of catalysis was suggested on the basis of high-resolution TcDH crystal structures and electronic paramagnetic resonance (EPR) spectroscopy, complemented with computational, enzymatic and mutagenesis studies [3]. TcDH is a functional homodimer, whose monomers are seven-bladed β-propeller superbarrels, with catalytic centers comprising three copper ions located in the central tunnels of the β-propellers (Figure S1A). The unique configuration of the metal cluster enables two-electron oxidation of thiocyanate ion to cyanate and elemental sulfur. As it was suggested in [3], thiocyanate ion binds in the active site of TcDH, forming coordination bonds by the nitrogen atom with the Cu1 ion, and by the sulfur atom with the Cu2 and Cu3 ions, while the C atom is located against the catalytic water molecule acting as a nucleophilic agent (Figure S1B). The C-S bond cleavage and C-O bond formation occur in a single step, followed by the oxidation of the reduced sulfur atom to elemental sulfur through two subsequent one-electron transitions to copper ion (Cu2 or Cu3), which is reduced to the oxidation state Cu^+^. Consequently, electrons are transferred from the Cu^+^ ion to the external electron acceptor. A horse heart cytochrome *c*550 (hhCytC550) and low-molecular-weight mediators were used as artificial electron acceptors since a second component of the TcDH-dependent electron transfer (ET) chain in *T. paradoxus* ARh 1 has not been identified. 

ET chains in the periplasm of SOB are an actively investigated and discussed field of research that significantly suffers from a limited amount of structural information about components of ET complexes. In general, ET complexes are transient with a half-life time on the millisecond timescale and have a low binding affinity (K_d_ in the micromolar to millimolar range) [10]. The transient nature, which distinguishes ET complexes from the more stable and long-living antigen–antibody, inhibitor–enzyme, or signal transduction complexes, facilitates rapid ET but complicates the isolation and characterization of the corresponding ET complexes. 

A protein with molecular weight (MW) of 25 kDa (according to the mobility in the SDS-PAGE), routinely copurified with TcDH from the periplasm of *T. paradoxus* ARh 1 growing in the presence of thiocyanate, was identified by MALDI-TOF mass-spectrometry as a single-heme cytochrome *c* (CytC552). Co-purification of the proteins from the same compartment of the bacteria suggests their putative interaction. Moreover, other lines of evidence point to the possible relationship between CytC552 and TcDH. The genes of CytC552 homologues were found in the genomes of all *Thioalkalivibrio* strains containing the *tcdh* genes (Supplementary Table S1). Transcriptom study of *T. thiocyanoxidans* ARh 2T (closely related to *T. paradoxus* ARh1) showed thiocyanate-dependent induction of the expression of both proteins TcDH (logFC 7.5, P 1.60E-17, [2]) and CytC552 homologue (logFC 8.3, P 2.2E-83, T. Berben, personal communication).

To clarify the putative interactions between TcDH and CytC552, we produced this cytochrome in *Escherichia coli*, evaluated its spectral and redox properties, analyzed TcDH activity in the catalytic reaction with CytC552 as an electron acceptor and confirmed the TcDH-CytC552 interaction by isothermal calorimetry (ITC). 

The spatial structure and conformational flexibility of oxidized CytC552 were determined by high-resolution nuclear magnetic resonance (NMR) spectroscopy supplemented with small-angle X-ray scattering (SAXS). High Ambiguity Driven protein-protein Docking (HADDOCK) [11,12] approach, based on NMR chemical shift perturbation (CSP) analysis, allowed us to predict the model of the CytC552-TcDH complex, while the ET pathway was suggested using Pathways plugin for VMD [13]. These findings represent the first step in elucidation of the TcDH-dependent ET chain in the periplasm of the thiocyanate-oxidizing bacteria of the genus *Thioalkalivibrio*.

## 2. Results and Discussion

### 2.1. CytC552 Is Characteristic of the Genus Thioalkalivibrio Strains Expressing TcDH and Serves as Its Electron Acceptor

CytC552 was copurified with TcDH from a periplasmic fraction of *T. paradoxus*. Using MALDI-TOF-MS (peptide fingerprint), the cytochrome was identified as the WP_006748979.1 protein, which is the THITH_RS18145 gene product. 

Genes of homologous cytochromes *c* were found in the genomes of all bacterial strains of the genus *Thioalkalivibrio*, which contained the *tcdh* genes (Table S1 and Figure S2A,B) [14]. In all the genomes, the genes of CytC552 homologues were located upstream of the thiocyanate dehydrogenase operon [2]. All homologous cytochromes from *Thioalkalivibrio* have a highly conserved core containing the C(V/L)RCH heme-binding motif and less conserved N- and C-terminal extensions (Figure S2A). 

Analysis of the CytC552 amino acid sequence showed that the protein has one heme-binding motif CVRCH, several His2-3 repeats at the N- and C-terminal termini, and 26 residues signal peptide (according to the SignalP 5.0 software (DTU Health Tech, Lyngby, Denmark)). These His-repeats are also found in the CytC552 closest homologue from the *T. nitratireducens* (Figure S2A). A calculated MW and isoelectric point (pI) of the mature protein were 18,101 Da and 5.87, respectively. MW of the protein, determined by MALDI-TOF-MS as 18,084 Da (Figure S1C), coincides with the calculated one. Location and sequence of the heme-binding motif indicate that CytC552 belongs to the class I that comprises soluble single-heme cytochromes *c* participating in ET processes in mitochondria and bacteria with the heme *c* attachment site (CXXCH) located about forty residues closer to the N-terminus than the sixth heme iron ligand (Met, His, or Cys) [15]. At the same time, the CytC552 polypeptide chain is longer than the one typical for the class I cytochromes *c* with MW around 12 kDa.

**Figure 1:**
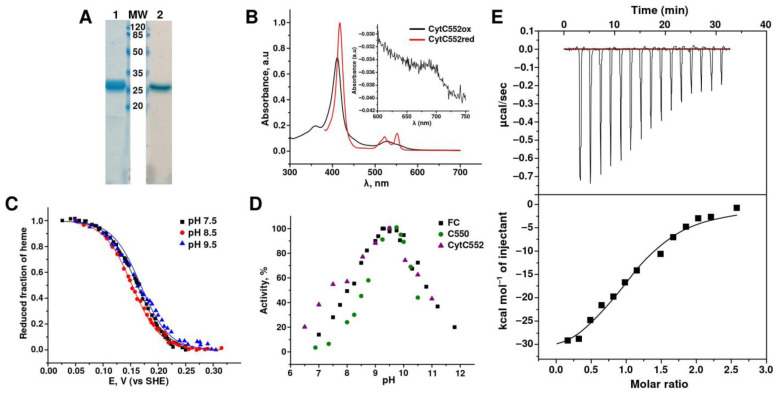
Characteristics of recombinant CytC552. (**A**) Assessment of homogeneity of CytC552 by 15% SDS-PAGE: 1-Coumassie Blue R staining, 2-heme *c* staining, MW-molecular mass (kDa) markers. (**B**) UV–visible spectra of the CytC552 oxidized form with characteristic peaks at 411 and 528 nm and reduced form with peaks at 417, 522, and 552 nm. (**C**) Redox titration of CytC552 at different pH values: 7.5, 8.5, and 9.5. The fraction of the protein reduced for each spectrum was calculated from A_552_–A_568_ (A_568_—absorption in an isobestic point). (**D**) pH-dependence of TcDH activity in the reaction of thiocyanate oxidation with CytC552, hhCytC550, and ferricyanide (FC) as electron acceptors. (**E**) Evaluation of the TcDH-CytC552 complex by isothermal titration calorimetry (ITC).

Using *E. coli* cells (BL21(DE3)), containing the pEC86 plasmid and vector plasmid for periplasmic expression, we obtained recombinant CytC552 in the heme-containing form. His-repeats at the N- and C-termini of the molecule were used for the protein purification by affinity chromatography on Ni-NTA agarose followed by size-exclusion chromatography. The SDS-PAGE analysis confirmed the purity of the isolated recombinant protein (Figure 1A). The CytC552 MW predicted from the electrophoretic mobility was about 25 kDa, which is higher than calculated MW (18 kDa). This phenomenon was reported for highly acidic proteins [16]. 

Both native and recombinant CytC552 exhibit typical visible spectral characteristics of cytochromes *c* with maxima at 411 and 528 nm for the oxidized form and at 417, 522, and 552 nm for the reduced form (Figure 1B and Figure S3A (comparison of two cytochromes)). A small maximum at 695 nm (see insert in Figure 1B) is characteristic of heme *c* coordinated by His and Met in axial positions [17,18]. Similar content of the heme fraction (A_411_/A_280_) in the native and recombinant CytC552 indicate that posttranslational processing of the recombinant protein has been successful. The high efficiency of heme incorporation was additionally confirmed by the pyridine method [19].

The enzymatic reaction of thiocyanate (SCN^-^) oxidation catalyzed by TcDH is accompanied by transfer of electrons to the acceptor. As shown in [3], hhCytC550 and low-molecular-weight mediators can serve as electron acceptors in vitro. To examine that the endogenous cytochrome CytC552 can perform this function, its redox potential was determined and the specific activity of TcDH in the reaction of thiocyanate oxidation with CytC552 as an electron acceptor was measured. 

Redox titration of CytC552 with control of α-band absorption at 552 nm at three different pH values 7.5, 8.5, and 9.5 revealed that the cytochrome is redox-active in the potential range of 250 ± 50 mV (Figure 1C). The reductive and oxidative curves are superimposable (Figure S3B), which indicates that under these experimental conditions CytC552 can reversibly cycle between the fully reduced and fully oxidized states. The E_m_ values for CytC552 (162.1 ± 0.5 mV at pH 7.5, 152.8 ± 0.5 mV at pH 8.5, and 164.1 ± 1.3 mV at pH 9.5) are almost independent of pH. Comparisons with previously used electron acceptors show that CytC552 has a lower redox potential than both hhCytC550 (E_m_ near 260 mV at pH 9.5) and ferricyanide (FC) (E_m_ near 340 at pH 7.0) [3].

To optimize the conditions for measuring the TcDH activity in the reaction with CytC552, the pH optimum of the reaction was determined to be 9.5 (Figure 1D). This pH value matches the pH of the host organism habitat. The same pH optimum of 9.5–10 was observed for other tested electron acceptors hhCytC550 and FC (Figure 1D). In the case of hhCytC550 and FC, pH-dependences are bell-shaped. Previous analysis of TcDH activity attributed pKa of the acidic and alkaline parts of the pH-dependence curves (8.5 and 10.5) to the protonation/deprotonation of essential His and Lys catalytic residues in the TcDH active site [3].

A characteristic feature of the pH dependence of the TcDH activity with CytC552 is the presence of a small shoulder on the acidic part of the pH-dependence curve (Figure 1D). The appearance of this shoulder may be due to the fact that CytC552 has a pI of 5.87 and is in an anionic form in the acidic pH region, which may affect its interaction with TcDH. Processing of the experimental curve with the introduction of an additional equilibrium for deprotonated CytC552 allowed us to calculate the pKa for the shoulder equal to 6.7, which is close the pKa of the NH^+^-group in the His imidazole ring. 

The specific activity of TcDH in the reaction with CytC552 measured at the pH-optimum 9.5 (Asp = 7.3 ± 1.1 µmol × min^−1^ × mg^−1^) is comparable to the activity previously obtained for hhCytC550 (Asp = 10.9 ± 1.3 µmol × min^−1^ × mg^−1^) and FC (Asp = 11.8 ± 0.8 µmol × min^−1^ × mg^−1^) [3]. Somewhat lower activity with CytC552 compared to other acceptors maybe accounted for its lower redox potential. The dissociation constant (K_d_) of the TcDH-CytC552 complex measured by the ITC was 1.8 ± 0.4 µM (pH 9.5) (Figure 1E). This affinity in the micromolar range indicates the possibility of the existence of the TcDH-CytC552 complex in the periplasm of *T. paradoxus*, which is consistent with the observed copurification of the proteins.

### 2.2. CytC552 Solution Structure Revealed Significant Heme Exposure to Solvent and Disordered N- and C-Terminal Extensions 

**Figure 2:**
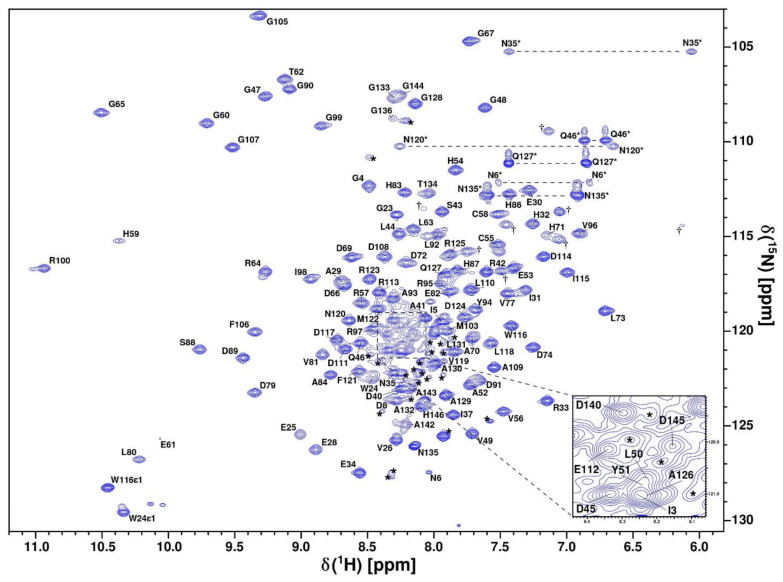
Assigned ^1^H-^15^N-HSQC spectrum of uniformly ^13^C/^15^N-labeled oxidized CytC552 recorded at 800 MHz resonance frequency, pH 7.0, and 303 K. Cross-peaks of side-chain NH_2_ groups of Asn and Gln are connected by dashed lines and marked by asterisks. Side-chain Hε–Nε correlation peaks of Arg are marked with dagger signs (†). Unassigned cross-peaks are indicated by asterisks.

**Figure 3:**
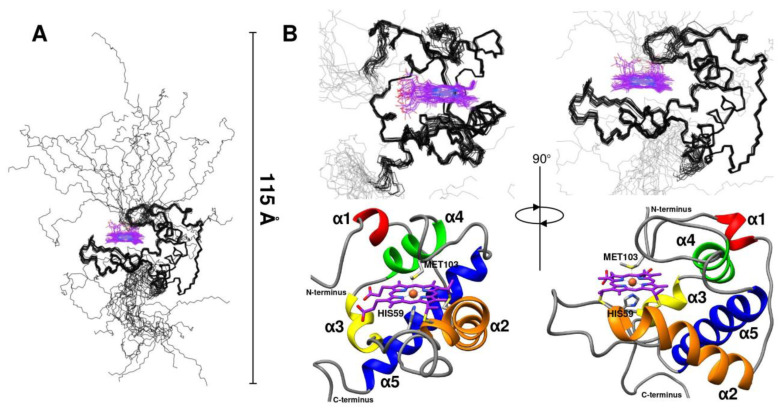
Solution NMR structure of oxidized CytC552. (**A**) Graphical representation of backbone overlay for the final ensemble of 20 structures (carbon atoms of the heme *c* group are colored in violet). Maximum size of polypeptide chain is indicated by scale bar. (**B**) Main-chain representation (in two projections) of globular part of NMR ensemble (top) and ribbon representation of lowest energy structure with the α-helices highlighted by contrast colors (bottom). Heme ligating Cys55 and Cys58 residues, and axial coordinating His59 and Met103 residues are shown with sticks.

The structure and dynamic properties of CytC552 were studied by heteronuclear NMR spectroscopy using uniformly ^13^C/^15^N-labeled recombinant protein. The chemical shift assignment was performed for the CytC552 oxidized state based on the analysis of the set of 2D and 3D heteronuclear NMR spectra (see Materials and Methods section). The CytC552 polypeptide chain consists of 153 amino acid residues, 126 of which have been assigned (Figure 2). The total number of assigned chemical shifts is 1312 from which 451 carbon atoms, 121 nitrogen atoms, and 740 hydrogen atoms. The overall assignment completeness for ^1^H, ^13^C, and ^15^N chemical shifts is 79%. The amide resonances of 26 unassigned residues: 9–22, 38, 75, 101, 137, 138, and 147–153, whose cross-peaks are indicated by asterisks in the central region of the spectrum (Figure 2), can be attributed to their increased mobility and/or higher water exchange rate, which is typical for an intrinsically disordered polypeptide chain (IDP). The obtained chemical shifts were also used to analyze the secondary structure and derive dihedral angles restraints using Talos-N [20]. The assigned chemical shifts were deposited into the BMRB database under the accession code 34618. The solution NMR structure of oxidized CytC552 on the final stage was calculated by XPLOR-NIH using 1907 distance restraints derived from NOE cross-peak intensities and 246 (φ and ψ) backbone dihedral angle restraints derived from chemical shifts analysis by Talos-N. Totally 100 structures were calculated, 20 of which with the lowest energy were selected for the final NMR ensemble (Figure 3) that was deposited into the protein structures databank (PDB) under the accession code 7O9U. The data on the assignment, restraints, and structural statistics for the NMR ensemble of CytC552 structures are given in Table S3. 

The CytC552 structure can be divided into two parts: a globular part (residues 24–132), which forms a stable α-helical core for the heme-binding pocket (average backbone RMSD for ensemble is 0.5 Å) and N- and C-terminal IDP regions (residues 1–23 and 133–153, respectively) attached to the core and enriched with histidine residues (Figure 3A and Figure S4). The structured globular part of the protein accounts for 71% of the total polypeptide chain length. It consists of five α-helices (α1–α5) and three loops (L1–L3) connecting the α-helices (Figure S4). The short α1-helix and the L1-loop are absent in the majority of classical class I cytochromes *c* (Figure S5). The longest helices α2 and α5 are located almost at right angles to each other. The heme-binding motif is in the C-terminal end of the α2-helix, followed by the L2-loop connecting it to the α3-helix, turning into the α4-helix (Figure 3B and Figure S4). The heme axial ligand (Met103) is in the L3-loop connecting the α4- and α5-helices. The characteristic feature of the CytC552 structure is significant exposure of the heme group to bulk solvent, which is associated with the small size of the L3-loop containing axial methionine (Figure 3B and Figure S5). In the majority of the class I cytochromes *c*, which demonstrates tremendous variability in both sequences and structures, neither the heme propionates, nor edges have any direct interaction with bulk solvent [15]. However, a group of structural homologues of CytC552 were revealed among bacterial cytochromes *c* using DALI Internet service [21] (Supplementary Figures S6 and S7).

A group of topological homologues of CytC552 consists of four unusually large cytochromes *c*551/552 (CytCL) from the periplasm of methylotrophic and denitrifying bacteria: *Me*-CytCL from *Methylobacterium extorquens* [22], *Hd*-CytCL from *Hyphomicrobium denitrificans* [23], *Pd*-CytCL from *Paracoccus denitrificans* [24], and *Ma*-CytCL from *Methylophaga aminisulfidivorans* [25] (Figures S6 and S7). All CytCLs are electron acceptors for pyrroloquinoline quinone-containing methanol dehydrogenases (PQQ-MDH) [22,23,24,25]. Similar to TcDH, PQQ-MDH belongs to the class of β-propeller enzymes with catalytic sites located in the central tunnels of the β-propellers [26,27]. Thus, both components of two donor-acceptor pairs PQQ-MDH/CytCL and TcDH/CytC552 possess topological similarity, which is a promising starting point for comparative analysis of ET chains in the periplasm of methylotrophic, denitrifying, and sulfur-oxidizing bacteria. 

PQQ-MDH catalyzes oxidation of methanol to formaldehyde passing two electrons through periplasmic ET chain, which includes CytCLs and small soluble cytochromes *c*2, to membrane-bound cytochrome oxidases [22,23,24,25]. Two models of putative PQQ-MDH/CytCL ET complexes were constructed using protein–protein docking [23] or homology modeling [24]. In the last case, quinohemoprotein alcohol dehydrogenase from periplasm of *Pseudomonas putida* (*Pp*-ADH) [28] served as natural prototype of ET complex between β-propeller-folded quinoprotein (as an electron donor) and CytCL (as an electron acceptor). The atomic resolution structure of *Me*-MDH confirmed the conservation of the active sites geometry and suggested a common ET pathway from PQQ to heme iron in *Pp*-ADH and *Me*-MDH/CytCL complex [27]. 

CytCLs share 50–55% identity between each other and 19–21% identities with CytC552 (Figure S6). Like CytC552, their large sizes are associated with N- and C-terminal extensions attached to the heme-containing α-helical scaffolds, which are similar to that of CytC552 (Figure S7A,B). At least one propionate and two edges of the CytCL hemes c are exposed to the solvent (Supplementary Figure S6C). However, according to the CytCLs crystal structures, their N- and C-termini contain additional alpha-helices and interact with the heme-containing cores, which is different from the IDP-like nature of the CytC552 terminal extensions. 

**Figure 4:**
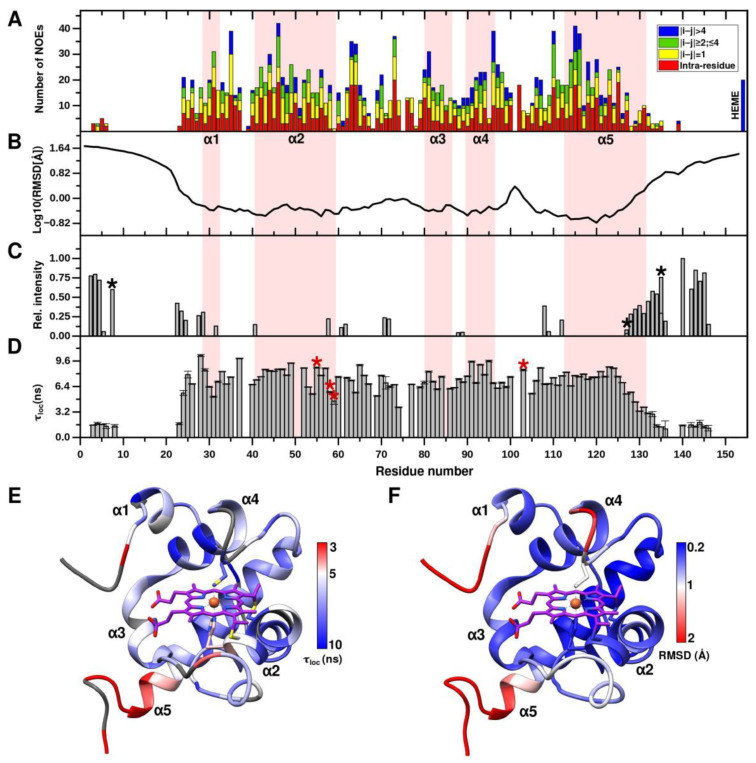
Summary of NMR structural and dynamical parameters of CytC552. (**A**) Distribution of NOE restraints used in structure calculation along the CytC552 amino acid sequence. (**B**) Heavy atoms RMSD plot for NMR ensemble of CytC552. (**C**) Intensities of cross-peaks of the residual amide groups from 2D ^1^H/^15^N spectrum of CLEANEX experiment. Side-chain NH_2_ groups are marked by black asterisks. (**D**) Distribution of local rotation correlation times (τ_loc_) of the CytC552 residues amide groups estimated from ^15^N CSA/dipolar cross-correlated transverse relaxation experiment. The heme-ligating and axial heme-coordinating residues Cys55/Cys58 and His59/Met103, respectively, are marked by red asterisks. (**E**,**F**) CytC552 ribbon representation color-codded according to the τ_loc_ and RMSD values, respectively.

It should be noted that there are almost no NOE peaks in NOESY-HSQC spectrum for N- and C-terminal extensions of CytC552. The absence of available NOE signals and corresponding restraints (Figure 4A) are closely related to the uncertainty of the conformation of the CytC552 N- and C-terminal regions (Figure 4B). To overcome this, we measured NMR parameters characterizing polypeptide chain water exchange and local dynamics. An intense exchange of amide protons of the assigned amino acid residues from the N- and C-termini with water molecules is revealed by analysis of cross-peaks intensities in the spectrum of CLEANEX experiment (Figure 4C). The IDP nature of the CytC552 N- and C-terminal regions is also confirmed by backbone NMR dynamics (Figure 4D). Local rotational correlation time (τ_loc_) for backbone amide groups, obtained from the cross- correlated transverse relaxation experiment, is significantly lower for the N- and C-termini compared to the globular core (Figure 4D,E). The average τ_loc_ for the globular part is 7.4 ns, while for the assigned amide groups of the N- and C-terminal IDP regions it does not exceed 2 ns. The τ_loc_ decreases from residue 25 towards the N-terminus and from residue 125 towards the C-terminus. In addition, the elongated L2- and L3-loops are quite flexible as revealed by the decreasing τ_loc_ values for backbone amide groups and enhanced RMSD values for the main chain atoms of the corresponding residues (Figure 4E,F). Summing up, all parameters presented in Figure 4 are sufficiently correlated and indicate the IDP nature of the N- and C-terminal extensions of CytC552. 

**Figure 5:**
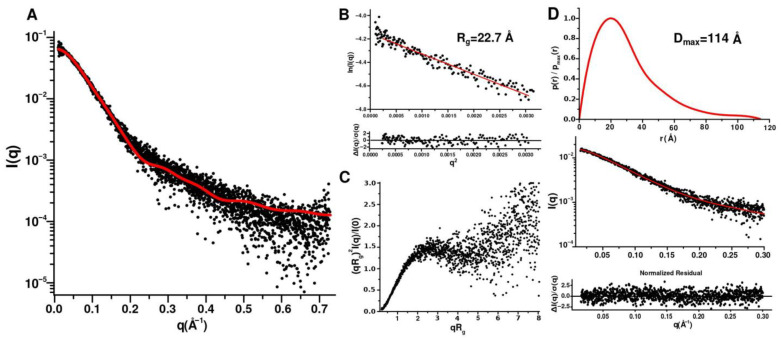
Analysis of SAXS data for oxidized CytC552. The measurements were carried out at 20 °C in 50 mM sodium phosphate buffer with pH 7.5. (**A**) Experimental SAXS profile (black circles) and theoretical curve (solid red line) calculated for the NMR structure of CytC552 using FOXS program. (**B**) Guinier plot with linear fit (red solid line). (**C**) Dimensionless Kratky plot. (**D**) Pair-distance distribution function profile with fitting curve (GNOM).

Moreover, the disordering of the N- and C-terminal regions is consistent with the data obtained by small angle X-ray scattering of CytC552 (Figure 5). The theoretical curve calculated for the NMR structure is in a good agreement with the experimental scattering profile (χ^2^ 0.28–1.03, depending on a structure from NMR ensemble) (Figure 5A). The radius of gyration (R_g_) for CytC552 is 22.7 Å according to the Guinier approximation of the initial Guinier region of the SAXS profile (Figure 5B), which is consistent with the size of the globular structured part of CytC552. The shape of the profile of the pair-distance distribution function p(r) (PDDF) (Figure 5C) and the peculiarities of the scattering curve in the Kratky coordinates (Figure 5D) indicate a significant content of disordered regions in CytC552. According to PDDF, the maximum protein size (D_max_) is 114 Å, which is in good agreement with the maximum size of the CytC552 molecule (115 Å) from NMR ensemble (Figure 3A).

### 2.3. Axial Heme Methionine Fluxionality of CytC552

**Figure 6:**
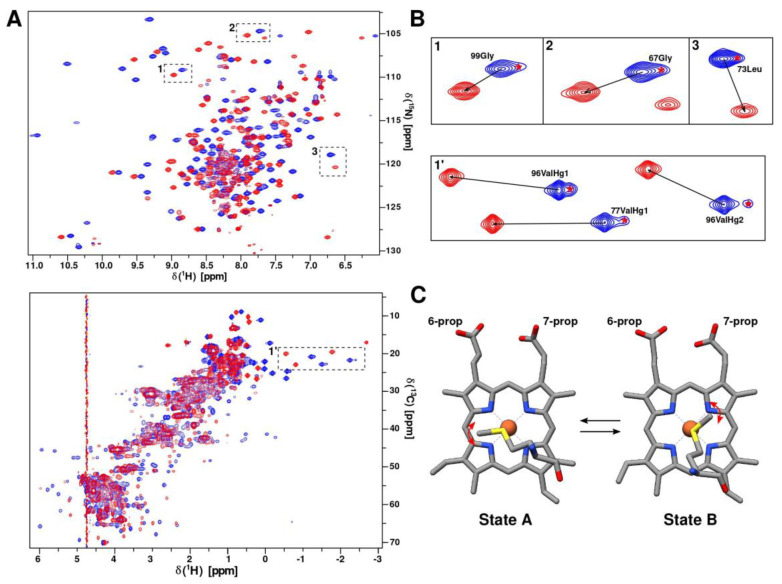
An axial heme methionine fluxionality. (**A**) Overlaid ^1^H/^15^N-HSQC (in top) and ^1^H/^13^C-HSQC-CT (in bottom) spectra of oxidized (blue) and reduced (red) forms of CytC552. (**B**) Selected regions of ^1^H/^15^N-HSQC (in top) and ^1^H/^13^C-HSQC-CT (in bottom) spectra with and without signal splitting for oxidized and reduced CytC552, respectively. The red asterisks indicate minor signals. (**C**) Two possible arrangements of heme-coordinating axial Met103 of CytC552 illustrating fluxionality (structures are taken from the NMR ensemble). Red arrows indicate methionine side-chain oscillation within a potential well of each state.

As can be observed in the ^1^H/^15^N-HSQC and ^1^H/^13^C-HSQC-CT spectra (Figure 6A,B), there is a splitting of a large number of cross-peaks into two states with unequal intensities. The splitting completely disappears in the CytC552 reduced state (Figure 6B). The splitting of the cross-peaks in the heteronuclear NMR spectra can be related to the change in the CytC552 paramagnetic properties due to fluxionality of axial heme-coordinating Met103 (rearrangement of the position of the Met side-chain relative to the heme plane). In the oxidized state (ferri-form), the Fe atom of the CytC552 heme is paramagnetic due to the presence of an unpaired electron. The unpaired electron creates a fluctuating magnetic field that affects the position of chemical shifts and the rate of nuclear relaxation of nearby atoms. The main effect of paramagnetism is the occurrence of pseudocontact chemical shift (*δ^pc^*^)^, which is described by the expression:

(1) $$\delta^{pc}=\frac{1}{24\pi }\frac{1}{r^{3}}\left\{\left[2\chi_{zz}-\left(\chi_{xx}+\chi_{yy}\right)\right]\left(3n^{2}-1\right)+3\left(\chi_{xx}-\chi_{yy}\right)\left(l^{2}-m^{2}\right)\right\}$$

and depends on the distance r and the position of the nucleus relatively to the principal axes of the magnetic susceptibility tensor (*χ_zz_, χ_xx_, χ_yy_*) [29]. Magnetic susceptibility tensor depends on the electronic environment of the paramagnetic center, i.e., on the structure of the complex of heme with ligands. Therefore, the anisotropy tensor of the magnetic susceptibility can change, when the heme axial ligand (Met103) undergoes conformational transition due to the fluxionality (Figure 6C) leading to the appearance of the pseudocontact shifts (Figure 6A,B). The signal splitting into major and minor peaks with the approximate intensity ratio is 6:1 indicating that one conformational state of the axial Met103 prevails over another. It is likely that the side-chain of the axial Met103 fluctuates in the region of the first state until the moment when it overcomes the activation energy barrier and moves into the second stable conformation. The absence of minor states for some cross-peaks near the paramagnetic center can be explained by the strong influence of paramagnetic relaxation, which leads to the broadening of minor signals below the detection limit. Fluxionality is supposed to require free space in the heme-containing pocket for the axial Met103 fluctuations, which is associated with the high SASA of the CytC552 heme group and the flexibility of the L2- and L3-loops surrounding the heme, revealed by increased RMSD and reduced local τ_loc_ values (Figure 4B,D). The analysis of the amide protons exchange in the main chain of hhCytC550 showed that the reduction of the heme iron causes the mobility decrease of the residues located in the heme-ligating region [30] leading to the assumption that a more mobile oxidized state favors interaction with a redox partner due to facilitation of the interface fitting by local structural rearrangements during ET complex formation.

Heme axial methionine fluxionality has previously been found for cytochrome *c*552 from *Hydrogenobacter thermophiles* by NMR line shape analysis [31]. Such process was not detected for the wild type of cytochrome *c*551 from *Pseudomonas aeruginosa*, while the introduction of the Asn64Gln mutation caused temperature-dependent heme methyl resonance line broadening, low rhombic magnetic anisotropy, and a change in magnetic axes orientation, which is consistent with the appearance of fluxionality for the axial methionine [32]. Fluxionality was associated with the appearance of free space due to Asn64Gln substitution favorable for the transition of the methionine side-chain group. The authors noted that for cytochromes *c* without fluxionality (for example, hhCytC550), dense packing is observed in the heme pocket.

### 2.4. NMR CSP Analysis of CytC552 Interaction with TcDH

**Figure 7:**
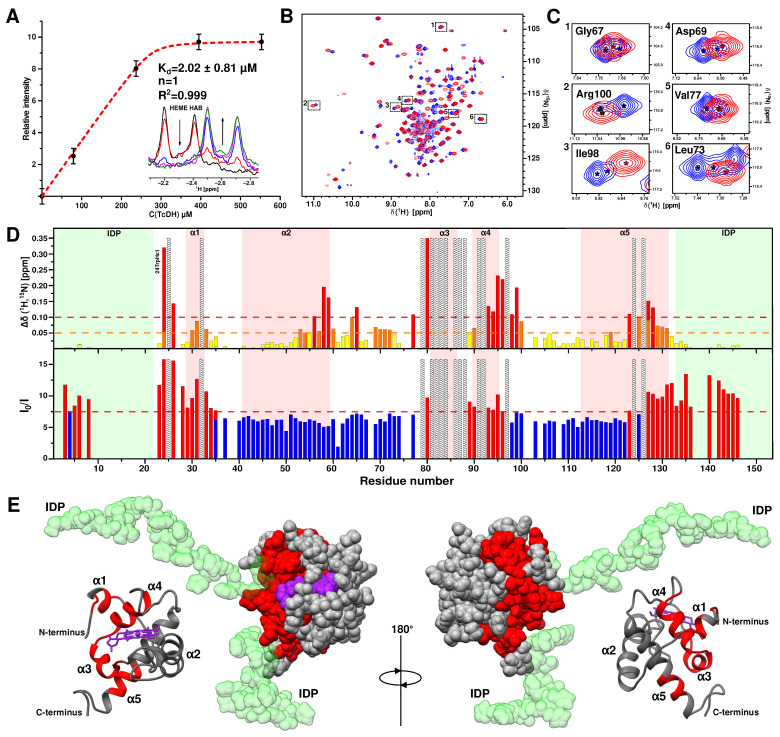
CSP analysis of the interaction of CytC552 with TcDH. (**A**) The dependence of the ^1^H-signal intensity of CytC552 heme group HAB atoms on the TcDH concentration. The red dashed line corresponds to the fitting curve. The inset shows the corresponding region of superimposed ^1^H spectra. (**B**) Overall ^1^H/^15^N-HSQC spectra of free CytC552 (blue) and bound to TcDH (red). (**C**) Selected regions of ^1^H/^15^N-HSQC spectra with cross-peaks with and without signal splitting for free CytC552 (blue) and bound to TcDH (red), respectively. (**D**) Histogram of generalized ^1^H/^15^N-chemical shifts changes for amide groups (top) and change of its cross-peaks intensity relative to unbound state of CytC552 (bottom). The residues, whose cross-peaks undergo either strong displacements (shifts) or broadenings beyond the detection range are shaded. The red dashed line corresponds to the standard deviation (SD) of the chemical shift changes and orange dashed line corresponds to the half of standard deviation (SD/2). The bars with values above the standard deviation are colored in red, in the range between SD/2 and SD are colored in orange, and below SD/2 are colored in yellow. On the graph of the cross-peaks intensity change (I_0_/I), the red dashed line corresponds to the average value (AV). The bars above the AV are colored in red and below are colored in blue. (**E**) Different types of CytC552 structure presentations colored according to the results of CSP analysis: the residues with highest CSP are highlighted in red; the N- and C-terminal IDP tails are colored in green.

**Figure 8:**
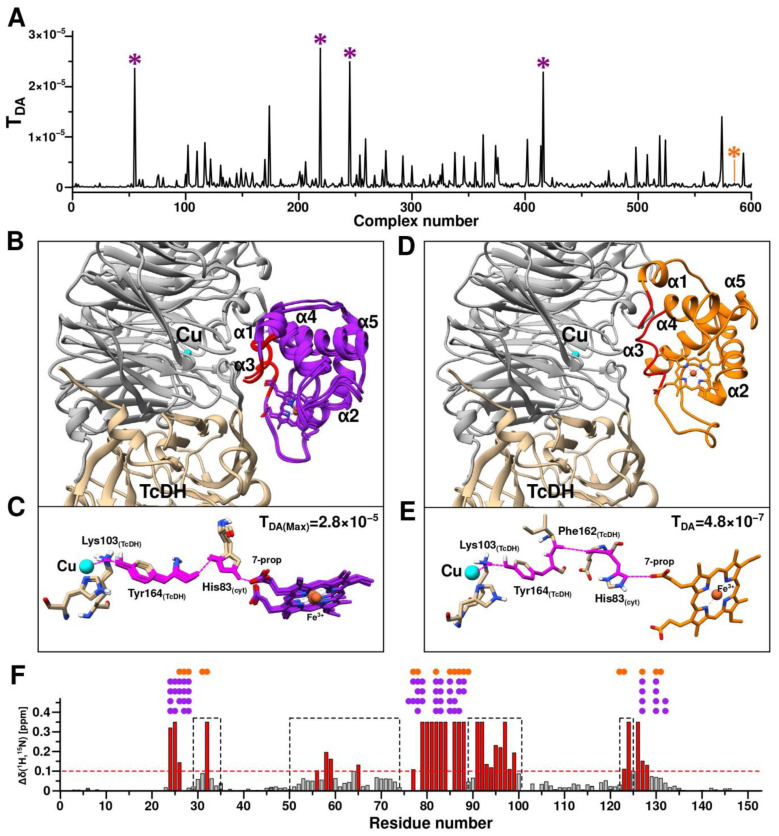
First stage of the TcDH-CytC552 complex structure prediction using HADDOCK (driven by ambiguous restraints (AIRs)) and Pathways program. (**A**) Plot of electron coupling constant (T_DA_) depending on structure number from HADDOCK calculations. Violet asterisks indicate the complexes with highest T_DA_ values and the orange asterisk marks the complex with the highest value of the HADDOCK scoring function. (**B**) Aligned structures of the four T_DA_-best TcDH-CytC552 complexes. TcDH monomers are colored in gray and light brown and CytC552 colored in purple. The exposed CytC552 residues that were used in AIRs generation in the HADDOCK calculations are colored in red. The Cu2 ion of TcDH is colored in cyan. (**C**) Electron transfer (ET) pathways predicted for the four T_DA_-best TcDH-CytC552 complexes using Pathways (pathcore) program. The ET pathways highlighted in pink: a solid line for transfer through covalent bonds and dotted line for transfer through space or hydrogen bond. (**D**) Haddock structure of TcDH-CytC552 complex with highest value of HADDOCK scoring function. (**E**) The ET pathway for TcDH-CytC552 complex with the highest value of HADDOCK scoring function. (**F**) The CSP histogram with marked residues of CytC552 that have intermolecular contacts with TcDH in complexes obtained by HADDOCK calculations (purple solid circles for complexes with highest T_DA_, orange for complex with highest scoring function value). Contacts are depicted as solid circles atop of corresponding residues. Areas highlighted with a black dashed line frame correspond to regions of the polypeptide chain with supposed allosteric effect on the CSP values. The red dashed line corresponds to standard deviation (SD) of the chemical shift changes; the residues having equal or higher values relatively SD are highlighted in red.

It is assumed that NMR signals of the CytC552 residues in contact with the TcDH surface demonstrate strong shifts and additional signal broadenings due to local increase in the transverse relaxation rate. To determine the interaction interface of the CytC552-TcDH complex, CSP analysis was performed (Figure 7). First, 1D ^1^H spectrum changes for strong field shifted protons of the heme group were analyzed upon CytC552 titration with TcDH (Figure 8A). The nature of the spectral changes (two separate states) indicates a slow exchange regime in the NMR time scale. The saturation is reached at the molar concentration ratio ~ 1:1 (CytC552:TcDH). The affinity of CytC552 to TcDH was determined using the chemical shift signal intensity change of HAB atom of the CytC552 heme group [33]. Equilibrium dissociation constant (K_d_) was estimated using dependence of the chemical shift signal intensity upon ligand concentration according to the Morrison equation [34] adapted to the binding analysis [33].

(2) $$\Delta I_{\mathrm{obs}}=\Delta I_{\max}\left\{\left(n{\left[P\right]}_{t}+{\left[L\right]}_{t}+K_{d}\right)-{\left[{\left(n{\left[P\right]}_{t}+{\left[L\right]}_{t}+K_{d}\right)}^{2}-4n{\left[P\right]}_{t}{\left[L\right]}_{t}\right]}^{\frac{1}{2}}\right\}/2n{\left[P\right]}_{t}$$

where $\Delta I_{\mathrm{obs}}\;$ is the NMR signal intensity change, $\Delta I_{\max}\;$ is the maximum intensity change upon saturation of the binding sites, ${\left[P\right]}_{t}\;$ and ${\left[L\right]}_{t}\;$ are the total concentrations of CytC552 and TcDH, respectively, n is the number of binding sites in the enzyme. NMR-derived K*_d_* (~2 μM) is consistent with the results of ITC experiment (Figure 1E).

At the saturating concentration of TcDH, there are significant shifts of the cross-peaks in the ^15^N-HSQC spectrum of the CytC552 oxidized state (Figure 7B). It should be noted that in the TcDH bound state, the splitting of the cross-peaks in the correlation spectra disappears similarly to the case of the CytC552 reduced form (Figure 7C), which can be explained by the increase of structural rigidity of the CytC552 heme-containing pocket and the loss of the axial methionine fluxionality. 

According to the CSP analysis of amide group cross-peaks in the ^15^N-HSQC spectrum (Figure 7D), the residues of CytC552 involved in the interaction with TcDH were mapped (Figure 7E). It was found that the largest CSP (greater than the standard deviation of 0.1 ppm) were observed for the residues: 24–26, 32, 58, 59, 65, 77, 79–84, 86–88, 93–99, 123, 124, and 126–128, which indicates that a perturbation involves the α1-, α3-, and α4-helices and the C-terminal parts of the α5-helix (Figure 7D,E). The maximal perturbation “density” is observed for residues 79–88. However, the changes in the positions of the cross-peaks of residues: 25, 32, 79, 81–84, 86–88, 91, 92, 97, 124, and 126, cannot be determined due to the signal shift or broadening beyond the detection limit, which can be attributed either to the site of direct contact or significant conformational rearrangement due to the allosteric effect. We suggest that perturbations for the residues 91–100 (the α4-helix) probably occur due to the latest. 

The formation of the complex also affects the intensity of the amide cross-peaks of CytC552 due to an increase of the rotational correlation time and the rate of nuclear relaxation. Analysis of the intensity changes in combination with CSP makes it possible to identify the regions which are not directly involved in the interaction with TcDH. Such regions include residues 37–76 and 103–122, where the change in the signal intensity is minimal and associated only with an increase in the nuclear transverse relaxation rate upon complex formation (Figure 7E). The CSP observed for these residues can be associated with the conformational rearrangement caused by the allosteric effect. Taking into account the results obtained, we suggest that the area of direct contact between CytC552 and TcDH includes residues 24–26, 79–88, and 126–128.

The N- and C-terminal IDP regions of CytC552 either do not interact specifically with TcDH or are in the fast dynamic exchange with the interaction site, since there are no significant changes in their chemical shifts. The changes in the intensity of signals of these regions can be explained by a decrease in the degree of freedom due to binding and the resulting steric restrictions. It should be noted that the size of the CytC552-TcDH complex exceeds 100 kDa, which usually does not allow acquiring of high-resolution NMR spectra. The observed modest signal broadening (resulting in the signal intensity decrease) of both the CytC552 core and its N- and C-terminal extensions implies that the complex is not tight and CytC552 has some degree of mobility. It can be assumed that the observed spectral properties are the result of an exchange between a tightly bound and an intermediate state. In the intermediate state, CytC552 is quite mobile relative to TcDH and its lifetime prevails over the tight complex in which electron transfer can occur. Thus, the spectrum of CytC552 saturated with TcDH reflects the equilibrium between the intermediate and tightly bound form.

### 2.5. CytC552-TcDH Complex Modeling and Electron Transfer Pathways Prediction

In order to determine the potential electron transfer pathway from TcDH to CytC552, we used the NMR experimental data on CSP as restraints for the complex modeling with the HADDOCK program [11,12]. The resulting set of complexes, ranked by the HADDOCK scoring function, was used as an input for the Pathways program to select a structure of complex with the effective ET pathways. To model the complex, we used the TcDH dimer (see Figure S1) and CytC552 truncated on the IDP-terminal regions of the polypeptide chain. According to the results of the CSP analysis, IDP tails do not have specific contacts with TcDH and may be excluded from the complex modeling. Moreover, truncation of CytC552 is necessary to prevent the accumulation of unproductive complexes at the rigid body docking stage due to the steric problem. 

Determination of the structure of the TcDH-CytC552 complex comprised two stages. At the first stage, the complex was modeled using ambiguous interaction restraints (AIRs) derived from the CSP analysis and the assumption that CytC552 binds to the area of the substrate access channel of TcDH, where the distance from the electron donor (Cu2/3 of the TcDH catalytic center) to the surface of the protein globule is minimal. Next, the resulting structures were evaluated according to the compatibility of their intermolecular contacts with NMR experimental data and the efficiencies of their ET pathways determined by the Pathways program. In order to enhance sampling of the structures with the most effective ET pathways, the distances between the atoms of key residues of the selected complexes with satisfying criteria were measured and used as unambiguous restraints at the second stage of the modeling. 

Surface-exposed residues of CytC552 (24–26 and 79–88) with the highest CSP and TcDH residues of the substrate access channel area were used for the generation of AIRs. After the first stage of modeling, 600 structures of the TcDH-CytC552 complex were calculated. For all these structures, the ET pathways and the corresponding coupling constants (T_DA_) were determined (Figure 8A). Four aligned structures of the complexes with the highest T_DA_ have the same architecture (Figure 8B). These structures share one electron transfer pathway from the Cu2 ion to the 7-propionate of heme group via Lys103 and Tyr164 of TcDH, and His83 of CytC552 (Figure 8C). The maximum T_DA_ value in this case is 2.8 × 10^−5^. For comparison, the structure of the complex with the maximum HADDOCK score (−147.9) (Figure 8D) has another ET pathway with T_DA_ value of 4.8 × 10^−7^ (Figure 8E). The decrease of the T_DA_ value by two orders of magnitude arises due to the local differences in the relative positions of Tyr164 of TcDH and His83 of CytC552. In neither case described above, intermolecular contacts between CytC552 and TcDH contradict the experimental NMR data (Figure 8F). 

Based on the data obtained, we assumed that the ET pathway involving Tyr164 of TcDH and His83 of CytC552 can be realized in TcDH-CytC552 redox system of *T. paradoxus*. Therefore, at the next stage of modeling we used unambiguous restraints derived from the structure of the complex with the highest T_DA_. The distances between the Cu2 ion and the oxygen of heme 7-propionate were utilized, as well as between the atoms of Tyr164 of TcDH and His83 of CytC552 for the restraint’s generation. In this case, the ET efficiency is considered as a more important criterion than the HADDOCK score value, which ranked structural models according to the energy gain, without consideration of the fact that ET can occur through an intermediate state. 

**Figure 9:**
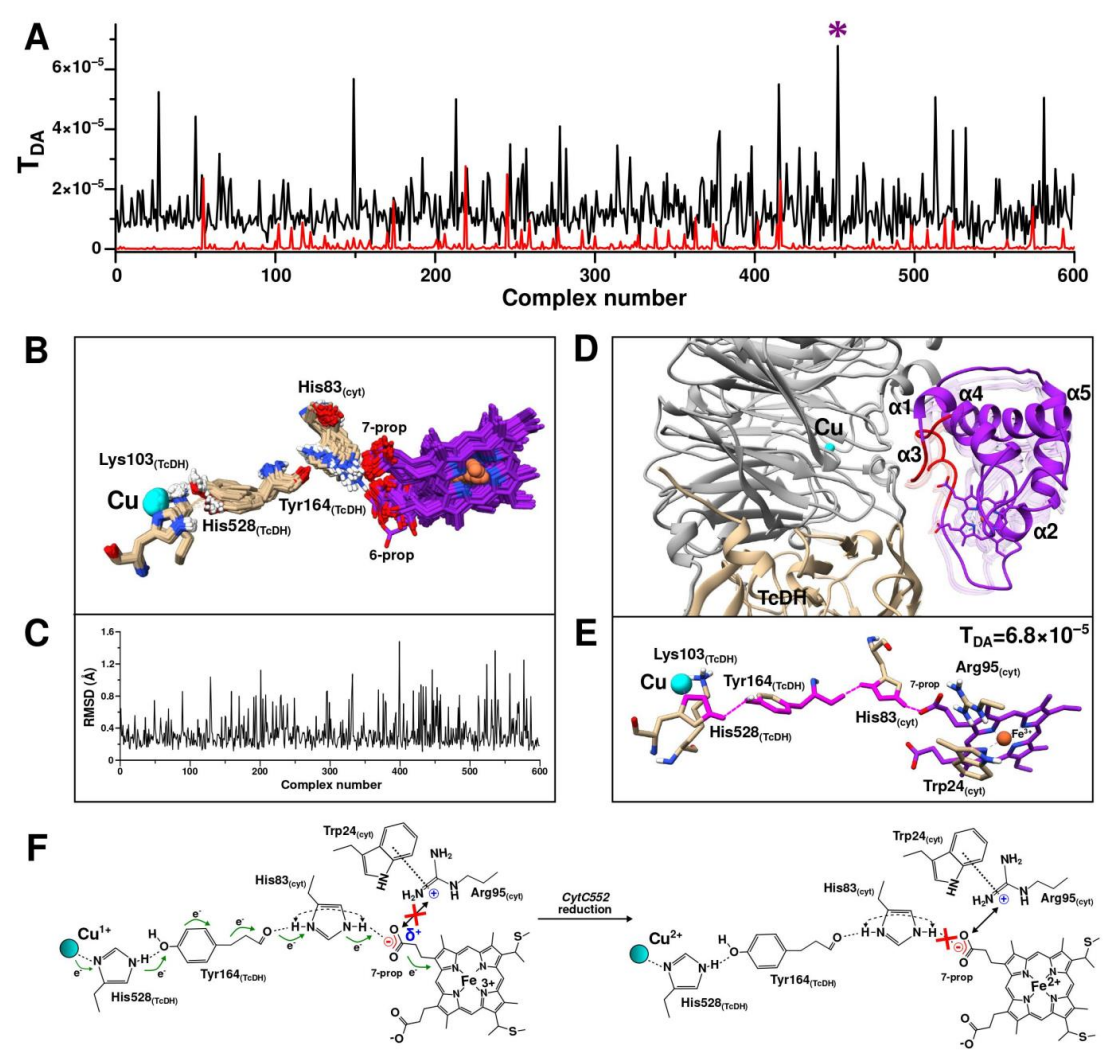
Second stage of the TcDH-CytC552 complex structure prediction using HADDOCK (driven by unambiguous restraints) and Pathways program. (**A**) Plots of electron coupling constant (T_DA_) depending on structure number from HADDOCK calculations with unambiguous restraints (black) and from the first stage of modeling with ambiguous restraints (red). Violet asterisks indicate the complex with highest T_DA_ values. (**B**) Superposition of structures of 600 HADDOCK complexes. Only the heme group and residues involved in ET are shown for clarity. (**C**) Plot of backbone atoms RMSD for all 600 structures relative to the average structure. (**D**) Structure of the complex with highest T_DA_ value. Superimposed structures from the first stage of modeling are shown as semi-transparent ribbon (from Figure 9B). (**E**) Predicted ET pathway (which started upon Cu2 ion reduction) for the complex with highest T_DA_ value. (**F**) Scheme of the electron transfer pathway between TcDH and CytC552. The black double arrow indicates a strong ionic interaction. The red cross on the left scheme indicates the absence of strong ionic interaction, and on the right scheme it indicates the absence of the corresponding hydrogen bond.

At the second stage of the HADDOCK modeling with unambiguous restraints, 600 structures of TcDH-CytC552 were also calculated and the ET pathways with the corresponding TDA values were predicted (Figure 9). All 600 structures were clustered into a single cluster. There was a significant increase in the TDA values for most structures compared to the first stage of modeling (Figure 9A). Alignment of all obtained structures revealed the high identity of the position of CytC552 relative to TcDH (Figure 9B), which was confirmed by backbone atoms RMSD (Figure 9C), as well as by the similarities of the ET pathways. There is only a variation of the ET pathway in the initial region depending on the position of Tyr164 of TcDH, which causes the transfer of an electron from the Cu2 ion to Tyr164 via either Lys103 or His528. The maximum value of T_DA_ is 6.8 × 10^−5^, which significantly exceeds the values from the first modeling stage. The corresponding complex is shown in Figure 9D in comparison with the complexes from the first stage of the HADDOCK simulation. The obtained results allow us to suggest an efficient ET pathway, which consists of an electron transition from the Cu2 ion to His528, then through space jump (or hydrogen bond) to the hydroxyl oxygen of Tyr164, followed by transition through the Tyr164 aromatic system to its peptide bond, then through space jump (or hydrogen bond) from the Tyr164 oxygen to the Hir83 side-chain group of CytC552 and finally jump to the carboxyl group of heme 7-propionate (Figure 9E).

It is worth noting the potential involvement of Trp24 and Arg95 residues of CytC552 in the electron transfer process (Figure 9F). We assume that an ionic bond is formed between positively charged Arg95 and negatively charged heme 7-propionate. The strength of such a bond depends on the oxidation state of the heme group. Thus, in the ferri-form, the partial charge on the 7-propionate group increases, which leads to destabilization of the electrostatic interaction with Arg95 side-chain but promotes the formation of the hydrogen bond between 7-prorionate oxygen and His83. In turn, Trp24 stabilizes the localization of the positive charge on Arg95 by the π-cation interaction, thus preventing the charge delocalization over the coupled electronic system. After heme reduction, the additional positive charge on the carboxylic group of 7-propionate disappears, which leads to the formation of a strong ionic bond between the latter and the Arg95 side-chain. For this reason, a hydrogen bond between 7-propionate and His83 of CytC552 cannot form, resulting in a significant reduction of the probability of an electron “leaking” in the opposite direction (to TcDH). All this contributes to efficient ET from the Cu2 ion of TcDH to the heme group of CytC552. 

It should be noted that the involvement of His83 of the CytC552 in the ET pathway from TcDH to CytC552 may explain the small shoulder in the acidic part of the pH-dependence curve of the TcDH activity discussed above (Figure 1D). At the pH below 6.7, His83 of the CytC552 is protonated in both positions of the imidazole ring, which contributes to the more efficient ET through a coupled system of hydrogen bonds (Figure 9F). At the pH above 6.7, His83 of the CytC552 is deprotonated and the curve of the pH dependence “slows down” as a result of disturbance of the “effective” electron density distribution. HhCytC550 does not have surface histidines that could be involved in ET, so there is no such shoulder in the corresponding curve (Figure 1D).

## 3. Materials and Methods

### 3.1. Purification of CytC552 from the Periplasmic Fraction of the T. paradoxus ARh1 Cells

The periplasmic fraction of the *T. paradoxus* ARh1 cells grown on thiocyanate was isolated as described in [3]. CytC552 purification involved three stages. The first step included anion-exchange chromatography on a MonoQ 10/100 GL column (GE Healthcare Life Sciences) pre-equilibrated with 25 mM MOPS, pH 7.5. The proteins were eluted with a 0–1 M linear gradient of NaCl in the same buffer. The CytC552-containing fractions were pooled and loaded on affinity HisTrap HP 5 mL column (GE Healthcare Life Sciences, Chicago, IL, USA) pre-equilibrated with the 50 mM Tris-HCl, 500 mM NaCl, 20 mM imidazole buffer, pH 8. Imidazole gradient from 20 to 500 mM in the same buffer was applied and fractions containing CytC552 were pooled, concentrated and loaded on the gel filtration column Superdex 75 10/300 GL (Amersham Biosciences, Chicago, IL, USA) pre-equilibrated with the 50 mM Tris-HCl, 150 mM NaCl buffer, pH 8. CytC552-containing fractions were analyzed by SDS-PAGE and MALDI-TOF-MS methods.

### 3.2. Production of Recombinant CytC552 

Cloning of the target gene, which coded the mature protein without 26 residues of the signal peptide, was performed according to standard procedures [35] with some modifications (see Supplementary Methods for details). *E. coli* strain Mach1 (Invitrogen) was used for cloning and strain BL21(DE3) (Novagen), co-transformed with plasmid pEC86 [36] was used for protein production. Plasmid pEC86 containing the *ccm* gene cluster, which improves the expression of mature *c*-type cytochromes in *E. coli*, was a kind gift from Dr. L. Thöny-Meyer (ETH, Zürich, Switzerland). 

Cultures were routinely grown in 2xYT medium (16 g/L tryptone, 10 g/L yeast extract, 5 g/L NaCl) or on 2xYT agar plates. Growth medium was supplemented with ampicillin (100 µg/mL) and chloramphenicol (34 µg/mL), where appropriate. The IPTG-induced overexpression of CytC552 in a rich medium (2xYT) was done according to [35]. 

For preparation of the uniformly ^13^C/^15^N-labeled recombinant CytC552, the BL21(DE3) transformed cells were cultivated in M9 minimal medium (6 g Na_2_HPO_4_, 3 g KH_2_PO_4_, 0.5 g NaCl per 1 L) that also contained 100 μg/mL ampicilline, 34 μg/mL chloroamphenicol, 2 mM MgSO_4_, 0.1 mM FeSO_4_, 1 mM δ-aminolevulinic acid (dALA), 0.001% thiamine chloride, and 0.0002% of yeast extract. The medium was supplemented with 0.2 mg/mL of ^15^NH_4_Cl (CIL, Tewksbury, MA, USA) and 2 g/L ^13^C-glucose (CIL, Tewksbury, MA, USA). The cells were cultivated at 37 °C to reach OD_600_ ~ 0.8. The protein expression was induced by 0.04 mM IPTG and continued for 16 h. 

Labeled and unlabeled CytC552 were isolated according to the same scheme. The cells were harvested by centrifugation at 4 °C and 5000× *g* for 15 min and periplasmic fraction was separated as described in [35] and loaded onto 5 mL HisTrap HP column (GE Healthcare, Chicago, IL, USA) equilibrated with binding buffer (50 mM Tris-HCl, 500 mM NaCl, 20 mM Imidazole, pH 8). Imidazole gradient from 20 to 500 mM in the same buffer was applied and fractions containing CytC552 were pooled, concentrated, and purified on HiLoad 16/60 Superdex 75 column (Amersham Biosciences, Chicago, IL, USA) equilibrated with 50 mM Tris-HCl containing 150 mM NaCl, pH 8. The chromatographic fractions were analyzed by UV–Vis spectroscopy and SDS-PAGE (15%). The gels either were stained with Coomassie R-250 or subjected to heme-staining procedure described in [37].

Spectrophotometric measurements were performed using spectrophotometer Cary 100 (Agilent Technologies, Santa Clara, CA, USA). Protein concentration was determined by the Bradford assay with bovine serum albumin (BSA) as a standard. The heme *c* concentration in purified protein and the extinction coefficient of CytC552 were determined by pyridine hemochromagen assay [19]. For the CytC552 preparation in fully reduced state, the sodium ascorbate was added to the final concentration of 5 mM.

### 3.3. Activity Assays

The standard TcDH activity was assessed as described in [3]. The reaction mixture contained 6 mM thiocyanate and 50 µM hhCytC550 (Sigma-Aldridge, St. Louis, MO, USA) as an electron acceptor in 25 mM borate buffer, pH 9.5. The enzyme concentration in the reaction was 10–20 nM for the Cu-saturated enzyme. The activity (µmol × min^−1^ × mg^−1^) was calculated from the steady-state rate of hhCytC550 reduction, which was measured spectrophotometrically at 550 nm (ε_550_ = 22.5 mM^−1^ cm^−1^) at 30 °C. 

When using recombinant cytochrome CytC552 as an electron acceptor, the reaction was carried out under the same conditions, but the rate of CytC552 reduction was measured spectrophotometrically at 552 nm (ε_550_ = 8.4 mM^−1^ cm^−1^). The concentration of TcDH in the reaction mixture varied from 10 to 100 nM. The activity was calculated as the average of three measurements. To determine the pH optimum of the reaction, 50 mM MOPS (pH 6.5–8) and 25 mM borate buffer (pH 8–11.5) were used.

### 3.4. Potentiometric Titration

Mediated spectrophotometric redox titration was performed as previously described [38]. The titration was carried out in an anaerobic glove box (Belle Technology, UK) under 100% N_2_ using platinum and Ag/AgCl electrodes in the presence of two mediators 2,6-dichlorophenolindophenol (E′_0_ +220 mV) and phenazine methosulfate (+80 mV) (10 µM each). Each redox titration was performed in both the oxidative and reductive directions using sodium dithionite and potassium ferricyanide solutions as the reductant and the oxidant, respectively. The degree of reduction was monitored by measuring A_552_-A_568_ (absorption at the wavelength of αmax for reduced CytC552 minus absorption at the isobestic point). The CytC552 concentration in a spectrophotometric cell was 13 µM. To determine the pH dependence of the redox potential, the following buffers were used: 50 mM HEPES, 150 mM NaCl, pH 7.5, and 25 mM borate, pH 8.5, and pH 9.5, both contained 150 mM NaCl. The reduction potentials were referenced to the standard hydrogen electrode. The titration curves were fitted by Nernst equation for one single-electron center.

### 3.5. Determination of the Dissociation Constant (K_d_) of the TcDH-CytC552 Complex by ITC

ITC experiments were carried out at 25 °C in 25 mM borate buffer, pH 9.5, using ITC 200 microcalorimeter (Malvern Panalytical, Malvern, PA, USA). Aliquots (2.6 µL) of CytC552 were injected into a 0.2-mL cell containing 9.3 µM TcDH solution to achieve a complete binding isotherm. CytC552 concentration in the cell changed in the process of the titration from 1.2 to 23.3 μM. The resulting titration curves were fitted using the MicroCal Origin-6.0 software, assuming either one or two binding sites of CytC552 in the TcDH dimer. Equilibrium dissociation constant (K_d_), enthalpy variations (ΔH), entropy variations (ΔS), and stoichiometry of binding (N) were determined based on Gibbs free energy (ΔG) equation.

### 3.6. NMR Spectroscopy 

NMR samples of uniformly ^13^C/^15^N-labeled CytC552 were prepared in 50 mM sodium phosphate buffer, pH 7.0, containing 5% D_2_O. Concentration of CytC552 was adjusted to 0.6 mM by ultrafiltration using 10 kDa molecular weight cut-off Amicon Ultra-15 Centrifugal filter units. For preparation of CytC552 in reduced state, the sodium ascorbate was added to 5 mM final concentration. The NMR spectra were acquired at 303 K using Bruker Avance III spectrometer with 600 MHz resonance frequencies for proton, equipped with a cryogenically cooled triple resonance 5 mm TCI probe with z-gradient and four RF channels. 

The ^1^H, ^13^C, and ^15^N chemical shifts of CytC552 were assigned by means of two- and three-dimensional heteronuclear experiments: ^1^H/^15^N-HSQC, ^1^H/^13^C-HSQC, ^1^H/^13^C-HSQC-CT (constant time version with evolution period of 28.6 ms), ^1^H/^15^N-TROSY, ^1^H/^15^N-HNHA, ^1^H/^13^C/^15^N-HNCA, ^1^H/^13^C/^15^N-HN(CO)CA, ^1^H/^13^C/^15^N-HNCACB, ^1^H/^13^C/^15^N-HNCO, ^1^H/^13^C/^15^N-HN(CA)CO, ^1^H/^13^C/^15^N-HBHA(CO)NH, ^1^H/^13^C/^15^N-(H)CC(CO)NH (with the mixing time of 12 ms), ^1^H/^13^C-HC(C)H-TOCSY (with the mixing time of 17 ms), ^15^N-edited TOCSY-HSQC (mixing time of 80 ms), and ^13^C- and ^15^N-edited NOESY-HSQC (with the mixing time of 100 ms). The ^13^C-edited NOESY-HSQC spectra optimized for aromatic and aliphatic groups were acquired separately. The backbone resonances of the peptides were assigned using the BEST-TROSY version of the triple resonance experiments [39]. The stability of CytC552 between long-time NMR experiments was monitored using the ^1^H/^15^N-HSQC and ^1^H/^13^C-HSQC-CT spectra. ^1^H and ^13^C chemical shifts were referenced to 3-(trimethylsilyl) propanoic acid, while ^15^N chemical shifts were calibrated indirectly. Proton chemical shifts of the heme *c* group were assigned using ^1^H/^1^H-NOESY (mixing time of 100 ms) and ^1^H/^1^H-TOCSY (mixing time of 80 ms) experiments recorded with enlarge ^1^H-spectral width of 22 ppm. NMR spectra were processed using TopSpin 3.2 (Bruker, Billerica, MA, USA) and NMRPipe [40]. Peak picking and chemical shift assignment were performed in CcpNmr Analysis v.2.5.1 (CCPN, Leicester, UK) [41]. The assignment of the backbone resonances was carried out using the manual approach and semi-automatic modes presented in CcpNmr. The chemical shift assignment correctness was checked manually twice. The obtained chemical shifts were used to analyze the secondary structure and local order parameters S^2^ of CytC552 using Talos-N [20]. In order to characterize the intramolecular dynamics, the effective rotation correlation times τ_loc_ were estimated for individual amide groups of CytC552 based on ^15^N CSA/dipolar cross-correlated transverse relaxation experiment acquired in interleaved fashion for the reference and attenuated spectra using a 2D ^1^H/^15^N-CT-TROSY-HSQC-based pulse sequence [42] with the constant period of 26.9 ms and the relaxation period of 10.8 ms. Generalized chemical shift differences, Δδ(^15^NH), for the amide groups are calculated as the geometrical distance (with weighting of ^1^H shifts by a factor of 5 compared to ^15^N shifts) between the amide cross-peaks assigned to the residues of free CytC552 and in its complex with TcDH. 

### 3.7. CytC552 NMR Solution Structure Calculation

In the first step of CytC552 spatial structure calculations, the dihedral angle restraints derived from the ^1^H, ^13^C, and ^15^N chemical shifts using Talos-N [20] and the distance restraints derived from the NOE (nuclear Overhauser effect) cross-peaks of acquired ^13^C- and ^15^N-edited NOESY-HSQC spectra were used. Automatic assignment of NOE cross-peaks and calculation of the preliminary structure of CytC552 without the heme *c* was carried out using ARIA 2.3 [43] in combination with CNS 1.21 [44,45] as an engine of torsion angles dynamics (TAD) calculations with the simulated annealing (SA) protocol. All data for ARIA2/CNS calculations were imported directly from the CcpNmr project. The structure calculations were performed taking into account the recommendations from [43,46]. A total of 1468 manually assigned NOE cross-peaks were used for the initial automatic assignment of all peaked NOE cross-peaks, as well as 246 dihedral angle constraints. The ARIA2/CNS calculation protocol, used in this work, is presented in Table S2. The calculations consisted of nine iterations, where each iteration was divided into a heating and two cooling stages. For the heating stage, 15,000 steps of the TAD with 27-fs step were calculated at the initial temperature 10,000 K and a final temperature 2000 K. The subsequent cooling stages were carried out in Cartesian coordinates. A total of 40,000 steps were used for the first and second cooling stages with final temperatures 1000 K and 50 K, respectively. All iterations were started from a random extended conformation. At each stage, 100 structures were calculated, 10 of which with the lowest energy values were used for the subsequent restraint violation analysis. In the last stage, the final 20 structures were refined using 40,000 steps of molecular dynamics in explicit water. 

The structure with the lowest total energy was selected for further refinement with heme *c* group. To obtain the preliminary structure of CytC552 with heme *c* group for further refinement, the energy minimization was performed using Amber 16 [47] with an initial steepest descent and subsequent conjugate gradient algorithm using ff14SB force field. In order to accomplish this, the heme *c* group was added to the CytC552 polypeptide chain manually using UCSF Chimera interface [48] taking into account the position of the coordinating residues Met103 and His59 and thioether covalent bonds between the CAC and CAB heme *c* atoms and the sulfur atoms of Cys55 and Cys58, respectively. The parameters for heme *c* were taken from the work [49] and adapted for heme *c* taking into account thioether bond formation based on the crystal structure of cytochrome *c*l from *Hyphomicrobium denitrificans* (PDB ID 2D0W) [23] and parameters from the work [50]. Amber-minimized CytC552 structure was used further for structure refinement with XPLOR-NIH 3.2 [51] using the SA protocol of TAD with subsequent minimization in explicit water. The structure calculations included 246 backbone dihedral angle restraints and 1907 NOE-derived distance restraints obtained from ARIA2/CNS calculations. A total of 20 restraints for the distances between heme *c* atoms and polypeptide chain atoms were obtained based on NOE cross-peaks from acquired 2D ^1^H-^1^H-NOESY spectrum. The SA protocol consisted of TAD stage with an initial temperature 3000 K and a final temperature of 25 K with cooling step of 12.5 K, as well as two successive stages of structure minimization in torsion angel and Cartesian spaces. The heme *c* structure was described using topology and parameters from topallhdg.hemes and parallhdg.hemes files included in XPLOR-NIH distribution. Parameters for two thioether bonds between heme *c* and Cys55/Cys58, and iron atom coordination by the His59 and Met103 residues were added by applying XPLOR patches for nonstandard PHCB, PHCC, PHMT, and PHEM residues, respectively. As a result, 100 structures were calculated, 20 of which with the lowest total energy were chosen into the final NMR ensemble and its quality was validated using PSVS [52] and wwPDB Validation Service [53].

### 3.8. SAXS Measurement and Analysis

Synchrotron SAXS measurements were performed at the European Molecular Biology Laboratory (EMBL) on the EMBL-P12 BioSAXS beamline at the PETRAIII storage ring (DESY, Hamburg), equipped with an automatic sampler and Pilatus 6M 2D photon-counting detector (DECTRIS, Switzerland). The scattering intensity I(q) was measured in the range of the momentum transfer 0.01 < q < 7.5 nm^−1^. Measurements were performed in 50 mM sodium phosphate buffer at pH 7.0 and temperature of 10 °C using a flow-through mode with a total exposure time of 1 s collected as 20 individual frames, each 50 ms, to monitor potential radiation damage. No radiation damage effects were detected. Data were corrected for the solvent scattering and processed using standard procedures. The SAXS measurements at three different protein concentrations were performed (0.4, 1.2, and 2.3 mg/mL) to determine possible influence of intermolecular interaction on resulting scattering profile. ATSAS 3.0.3 (EMBL, Hamburg, Denmark) [54] and BioXTAS RAW 2.1.1 [55] programs were used for processing and analysis of SAXS data. Radius of gyration and total forward scattering at zero angle I(0) were calculated using the Guinier approximation. The pairwise distance distribution function and the maximum particle size (D_max_) were determined using GNOM [56]. The flexibility and/or degree of unfolding of the protein globule was analyzed using a Kratky volume-of-correlation (Vc)-based plot [57]. The theoretical scattering curve for the NMR structure was calculated using the FOXS program [58].

### 3.9. HADDOCK Modeling of the TcDH-CytC552 Complex and Electron Transfer (ET) Pathways Prediction

Knowledge-based protein–protein docking method was used to determine the structure of the TcDH-CytC552 complex using GURU interface on the HADDOCK 2.4 webserver [11,12]. For the docking, the dimer molecule of TcDH from crystal structure 6UWE was used and the first structure from the CytC552 NMR ensemble, in which the flexible N- and C-terminal IDP regions (1–23 and 133–153 residues, respectively) were removed to prevent the steric problems leading to unproductive complexes at the stage of rigid-body modeling. For the first stage of modeling active ambiguous interaction restraints (AIRs) were used to drive docking process. AIRs were based on the CytC552 residues involved in the interaction according to the NMR CSP analysis and the TcDH surface residues within 10–15 Å from substrate access channel, close to the location of Cu2/3 ions of the active center. HADDOCK runs were performed with 50% random exclusion of AIRs in each structure calculation. The definition of flexible segments of neighboring residues was set to 5.0 Å. 

For the second stage of the modeling, unambiguous distance restraints derived from TcDH-CytC552 complex with the best ET pathway obtained on the first stage of HADDOCK modeling were used. Haddock modeling consisted of three stages: first, 3000 structures were calculated by rigid body docking, then 600 structures with best HADDOCK score were selected for semi-flexible refinement by torsion angle dynamics, which were consequently refined with an explicit solvent in Cartesian space and further analyzed using cluster analysis. The Fraction of Common Contacts (FCC) method was used for cluster analysis. For ambiguous restraints, energy constants were set to: hot–10, cool1–10, cool2–50, cool3–50 kcal/mole; for unambiguous restraints: hot–30, cool1–30, cool2–100, cool3–100 kcal/mole. The number of MD steps during second cooling stage with flexible side-chains at interface and number of MD steps during third cooling stage with fully flexible interface was increased to 3000. For final solvated refinement, the number of steps for heating phase was increased to 200, for 300 K phase to 2500, and for cooling phase to 1000. For non-bonded parameters, OPLSX force field was used. Electrostatic interaction was included during rigid body docking at all iterations (it0, it1).

The Pathways plugin [13] for VMD program [59] was used to identify dominant ET pathways for TcDH-CytC552 complexes, generated by HADDOCK, and estimate the donor-to-acceptor electronic tunneling coupling. PSF structure files were generated by standard autopsf VMD plugin. The analyzed complexes were matched using the GROMACS subprogram trjconv (2021.4 release) [60]. To process a large array of structures from HADDOCK calculations, a homemade bash script was used for automation. The Cu2 ion of TcDH was identified as the electron donor and the heme group of CytC552 as the acceptor.

## 4. Conclusions

Chemolithoautotrophic sulfur-oxidizing bacterium *Thioalkalivibrio paradoxus* ARh 1 can grow on thiocyanate as the only source of nitrogen and electrons due to the activity of TcDH, which catalyzes the thiocyanate oxidation to cyanate and elemental sulfur with two subsequent one-electron transitions to the external electron acceptor. In this work, unusually large single-heme CytC552, which co-purifies with TcDH from the periplasm of *T. paradoxus*, was produced in *E. coli* cells and tested as an electron acceptor in the TcDH-dependent thiocyanate oxidation. The specific activity of TcDH in the reaction with CytC552 was comparable to that previously obtained for exogenous electron acceptors. Moreover, the dissociation constant of the corresponding TcDH-CytC552 complex measured by both ITC and NMR-titration was in micromolar range (near 2 mkM), indicating high probability of the productive interaction. The solution structure and conformational flexibility of uniformly ^13^C/^15^N-labeled CytC552 in the oxidized state was determined by heteronuclear NMR spectroscopy supplemented with SAXS. The interaction interface between CytC552 and TcDH was mapped by CSP and used for the complex modeling by an information-driven docking approach. 

The structure-dynamic properties of CytC552 appear to be quite different from both classical (small) class 1 bacterial and mitochondrial cytochromes and CytC552 topological homologues—large cytochromes *c* from methylotrophic and denitrifying bacteria, which serve as electron acceptors for pyrroloquinoline quinone-containing methanol dehydrogenases, which in turn possess topological similarities with TcDH. We have found that, unlike all other cytochromes *c* from both bacteria and eukaryotes, CytC552 contains disordered N- and C-terminal extensions enriched with histidine residues. The role of such IDP extensions remains unclear, but the results of NMR measurements, particularly, the degree of broadening of signals in the ^15^N-HSQC spectrum of CytC552 in the complex do not correspond to what is expected during the formation of a tight complex with the size over 100 kDa. Thus, the observed signals in the ^15^N-HSQC spectrum are the result of an exchange between the tightly bound complex, in which ET occurs, and an intermediate one, in which CytC552 is quite mobile relative to TcDH. The lifetime of the intermediate state prevails over that of the tight complex. Apparently, the mobility of CytC552 in the intermediate state could be provided by non-specific interaction of the IDP extensions with TcDH.

In addition, the splitting of cross-peaks in the ^15^N-HSQC spectrum obtained for the CytC552 oxidation state indicates fluxionality of the heme axial methionine. Fluxionality is presumably associated with the increased loop dynamics near the heme group and high exposure of the heme to the solvent. The role of fluxionality remains a matter for further research, but we found that the cross-peaks splitting disappears upon interaction with TcDH. This behavior may be associated with the loss of fluxionality and the transition of CytC552 to a minor conformation.

Finally, in this work, an original approach was applied to predict the structure of the TcDH-CytC552 productive complex. This was achieved by filtering the complexes generated by HADDOCK based on CSP data through the Pathways program, which determines the best ET pathway. As a result, a relatively short putative ET pathway from TcDH to CytC552 was proposed, in which His83 of CytC552 may play one of the key roles. This finding may explain a small shoulder in the acidic part of the pH-dependence curve of the TcDH activity in the presence of CytC552. 

## Data Availability

The assigned chemical shifts were deposited into Biological Magnetic Resonance Bank (BMRB, https://bmrb.io/) under the accession code 34618. The final NMR ensemble was deposited into Protein Data Bank (PDB, https://www.rcsb.org/) under the accession code 7O9U.

## Supplementary Materials

The following supporting information can be downloaded at: https://www.mdpi.com/article/10.3390/ijms23179969/s1.
