# Supplementary material for: Unusual Cytochrome c552 from Thioalkalivibrio paradoxus: Solution NMR Structure and Interaction with Thiocyanate Dehydrogenase
Source: Int J Mol Sci. 2022 Sep 1;23(17):9969. doi: 10.3390/ijms23179969 (PMC9456337; doi:10.3390/ijms23179969)
Supplement: Supplementary file 1 [file ijms-23-09969-s001.zip › ijms-1881253-supplementary.pdf]

Supplementary data

UUnusual Cytochrome c552 from *Thioalkalivibrio paradoxus*: Solution NMR Structure and Interaction with Thiocyanate Dehydrogenase

Vladimir V. Britikov [1,†] , Eduard V. Bocharov [2,3,*,†] , Elena V. Britikova [1], Natalia I. Dergousova [4],
Olga G. Kulikova [4], Anastasia Y. Solovieva [4], Nikolai S. Shipkov [4], Larisa A. Varfolomeeva [4] ,
Tamara V. Tikhonova [4], Vladimir I. Timofeev [2,5], Eleonora V. Shtykova [5] , Dmitry A. Altukhov [6],
Sergey A. Usanov [1], Alexander S. Arseniev [2], Tatiana V. Rakitina [2] and Vladimir O. Popov [4]

[1] Institute of Bioorganic Chemistry, National Academy of Sciences of Belarus, 220141 Minsk, Belarus
[2] Shemyakin-Ovchinnikov Institute of Bioorganic Chemistry, Russian Academy of Sciences, Moscow 117997, Russia
[3] Moscow Institute of Physics and Technology, Dolgoprudny 141700, Russia
[4] Bach Institute of Biochemistry, Federal Research Center “Fundamentals of Biotechnology”, Russian Academy of Sciences, Moscow 119071, Russia
[5] Federal Scientific Research Center “Crystallography and Photonics”, Russian Academy of Sciences, Moscow 119333, Russia
[6] National Research Center “Kurchatov Institute”, Moscow 123182, Russia
* Correspondence: edvbon@mail.ru
† These authors contributed equally to this work.

Supplementary Tables

Table S1. *CytC552* homologues from bacteria of the genus *Thioalkalivibrio* containing *tcdh* genes.

Accession	Description	Identity, %	Query cover, %
WP_006748979.1	[Thioalkalivibrio paradoxus]	100	100
WP_015259500.1	[Thioalkalivibrio nitratireducens]	90	82
WP_156820921.1	[Thioalkalivibrio sp. AKL11]	64.5	82
WP_081616874.1	[Thioalkalivibrio thiocyanoxidans]	59.8	57

Table S2. Experimental restraints and structural statistics for the final NMR ensemble of *CytC552*.

**Completeness of resonance assignment**	**assigned/available (%)**
Total number of assigned shifts	1312 (79.0%)
All N	121/235 (51.5%)
All H	740/1069 (69.2%)
All C	451/707 (63.8%)
Backbone (N+H)	226/291 (77.7%)
Side-chain H	354/521 (67.9%)
Hα	139/172 (80.8%)
Hβ	183/239 (76.6%)
Hγ	95/119 (79.8%)
Hδ	69/108 (63.9%)
C'	113/153 (73.9%)
Cα	124/153 (81.0%)
Cβ	105/134 (78.4%)
All residues	126/153 (82.4%)
**NMR distance and dihedral constraints**	
**Distance restraints**	
Total NOE	1907
Intra-residual	735
Sequential (|i–j| = 1)	622
Medium-range (2≤ |i–j| ≤4)	321
Long-range (|i–j| >4)	229
Hydrogen bonds	0
Dihedral angle restraints (φ + ψ)	246
**Structure statistics**	
Structure calculated	100
Ensemble of lowest energy structures	20
**Restraints Violations**	
Distance constraints > 0.5 Å (mean ± s.d.) (Å)	6.6 ± 2.1
Dihedral angle constraints > 5° (mean ± s.d.) (°)	0
Max. distance constraints violation (Å)	1.3
Max. dihedral angle violation (°)	2.1
**Deviations from idealized geometry**	
Bond lengths (Å)	0.013
Bond angles (°)	1.6
Number of close contacts	0
**Ramachandran plot (Prochek)**	
Most favored region (%)	83.1
Additionally allowed (%)	16.8
Generous allowed (%)	0.1
Disallowed (%)	0.0
**Average pairwise r.m.s.d. (Å) (residues 23-132)**	
All Heavy	0.9 (PSVS)
All Backbone	0.5 (PSVS)
**Global quality scores (Raw score / Z-score)**	
Verify3D	0.22/-3.85
ProsaII	0.47/-0.74
Procheck (phi-psi)	-0.63/-2.16
Procheck (All)	-0.97/-5.74
MolProbity clash score	25.01/-2.77

Table S3. Parameters of the ARIA2/CNS 1.21 calculation protocol**.**

Parameter	value
Frequency window (proton1/2)	0.04/0.02
Frequency window (hetero1/2)	0.5/0.5
Trust assignments	No
Use only assigned	No
CNS topology file	topallhdg5.3.pro
CNS parameter file	parallhdg5.3.pro
Number of structures	60 (100)
Violation tolerance	15/4/4/2/2/2/1/1/0.5
Violation threshold	0.5
Ambiguity cutoff	-
Maximum nb. of contributions	6-10
Number of lowest energy structures	10 (20)
Solvent for refinement	Water
High temperature (tad)/ steps/ force constant	10000K/15000
High temperature (cartesian)	2000K/15000
Cooling 1 (cartesian)/ steps/ force constant	1000K/40000
Cooling 2 (cartesian)/ steps/ force constant	50K/40000
Refine step	40000
Log-Harmonic potential/ force constant	-
Sort criterion	Total energy
Number of iterations	0-8 + water refinement
Force constant for dihedral restraints	5/15/50/100
Force constant for distance restraints	5/5/8/10
Time step	0.003 ps
TAD time step factor	9 (tad-27 fs)

Supplementary Figures

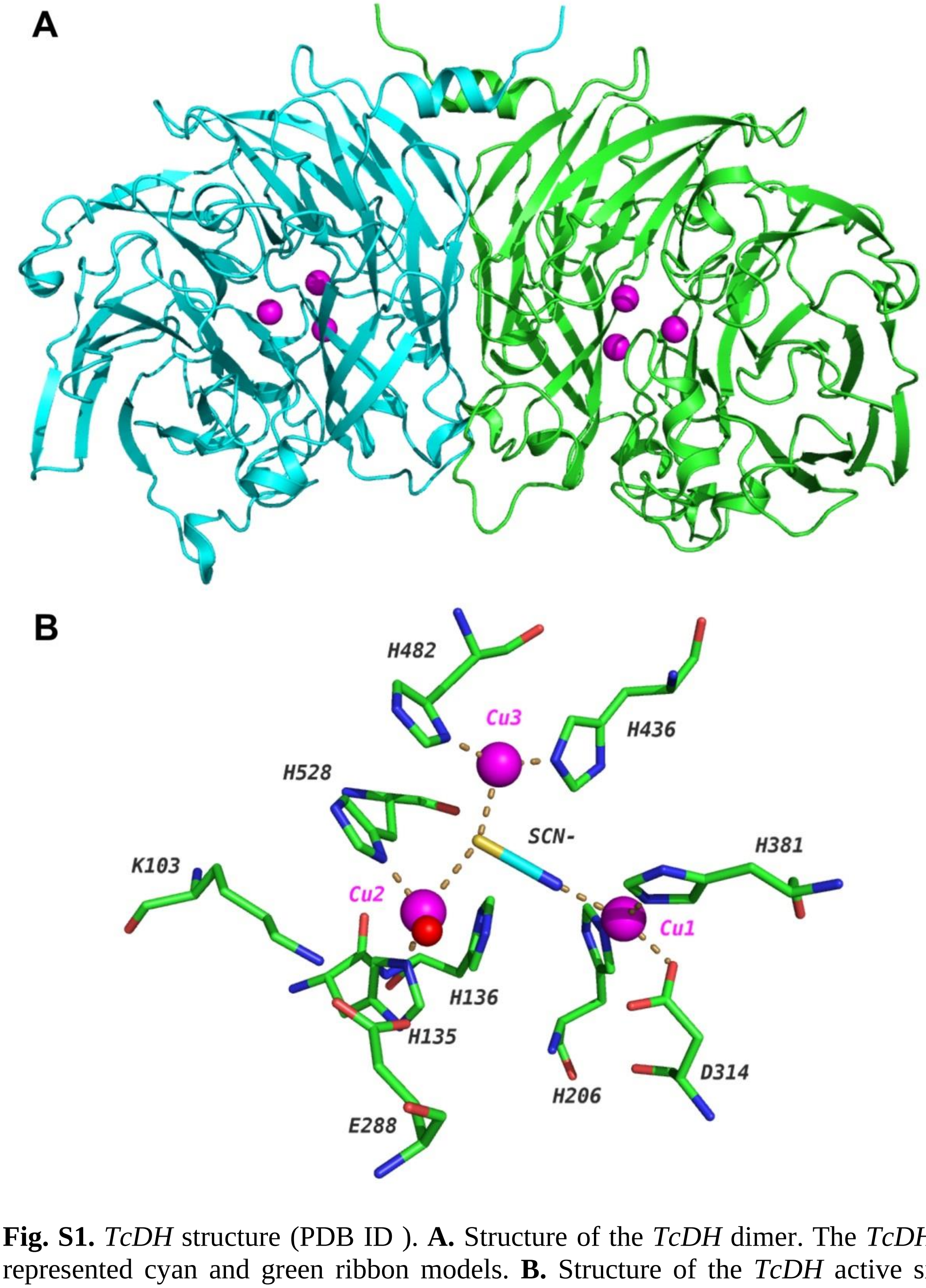

Fig. S1. *TcDH* structure (PDB ID). **A.** Structure of the *TcDH* dimer. The *TcDH* monomers are represented cyan and green ribbon models. **B.** Structure of the *TcDH* active site. The protein residues and thiocyanate ion are represented as ball-and-stick models colored by atom type. Copper ions (Cu1, Cu2, and Cu3) are shown as magenta spheres. Water molecules are shown as red spheres. The coordination bonds are indicated by dashed lines.

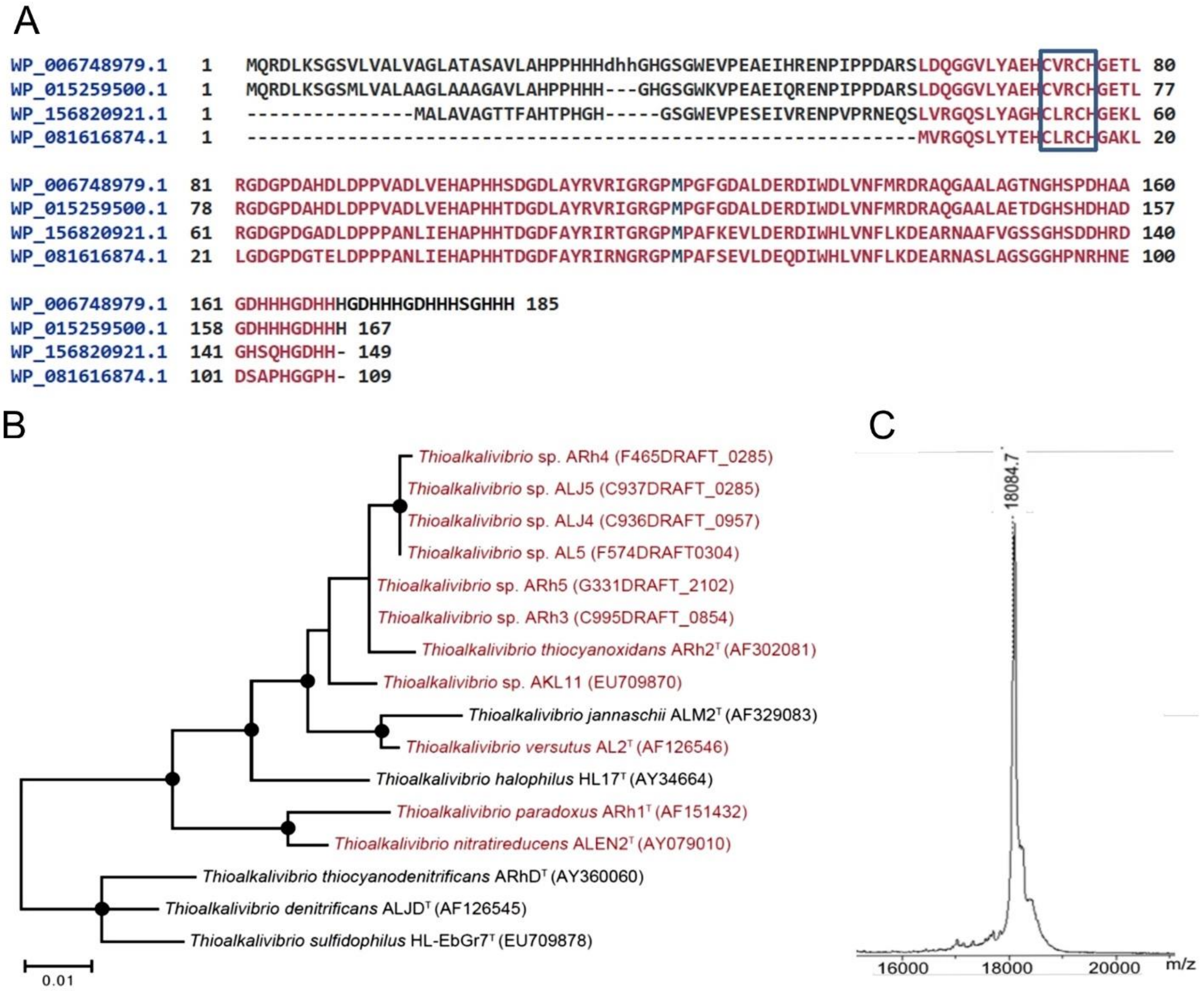

Fig. S2. *CytC552* (WP_006748979) and its homologues from bacteria of the genus *Thioalkalivibrio* containing *tcdh* genes. **A.** Amino acid alignment: α-helical cores are colored in burgundy, heme *c* binding motives CXXCH are in a blue box, Met residues coordinating heme in distal positions are colored in blue. **B.** Phylogenetic tree of bacteria of the genus *Thioalkalivibrio*: bacteria containing *TcDH* genes are highlighted in burgundy. **C.** Mass spectrum of full-size *CytC552*.

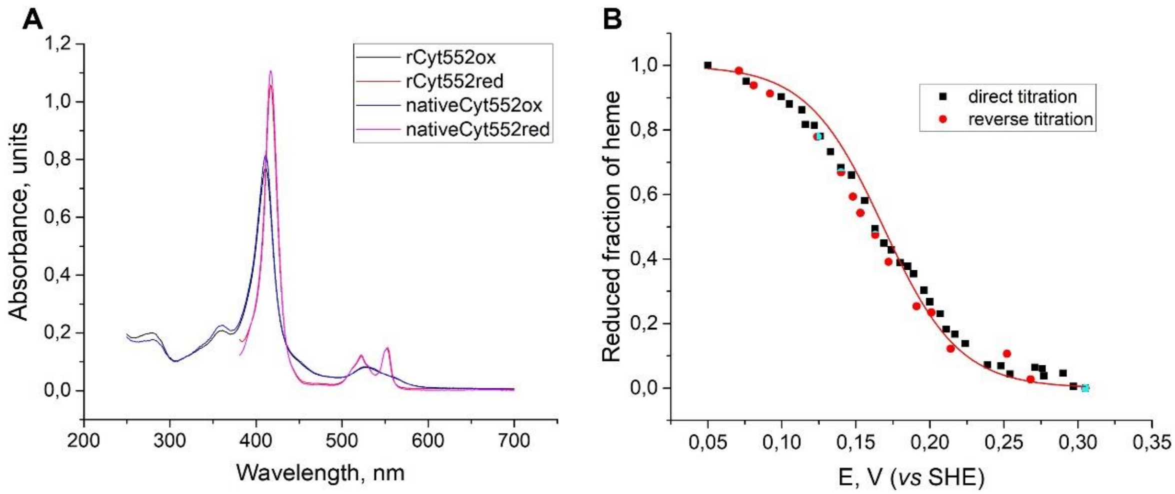

Fig. S3. Spectral and redox properties of the native and recombinant *CytC552*. **A.** UV-Vis spectra of oxidized and reduced forms. The A411/A280 ratios for the homogeneous proteins were 4.6 (native) and 3.8 (recombinant). **B.** Direct and reverse titration of *CytC552* at pH 9.5. Reduction potentials were referenced to standard hydrogen electrode (SHE). The fraction of the heme reduced for each spectrum was calculated from the difference A_{552}-A_{568}, where A_{568} - absorption in an isobestic point.

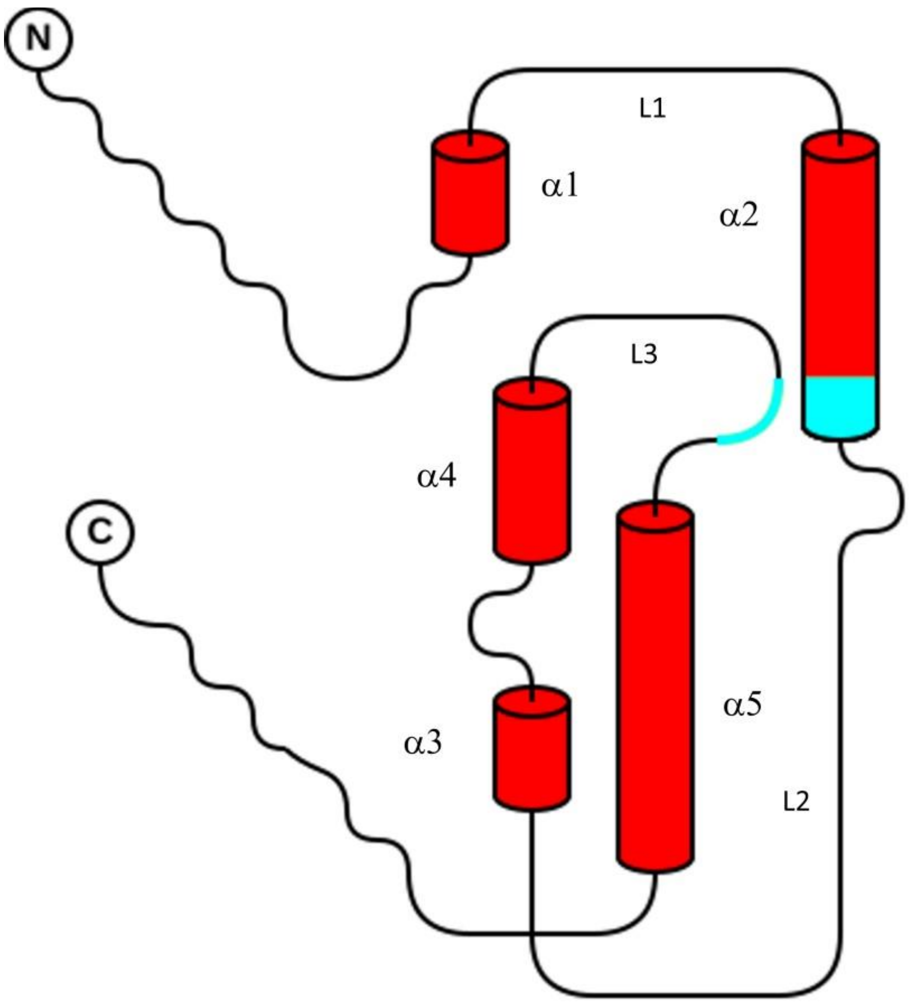

α1 L1 α2

^{1} MDIGINSDP^{10}HPPHHHDHHGHGS**GWEVPEAEIHRE**NPIPPDARSLDQGGVLYAEHCVRCHG^{60}

L2 α3 α4 L3

^{61}ETLRGDGPDAHDLDPPVAD**LVEHAPHHSDGDLAYRVRIGR**GPMPGFGDALDERDIWDLVNF^{121}

α5

^{122}M**RDRAQGAALA**GTNGHSPDHAAGDHHHGDHHH^{153}

Fig. S4. Topological scheme of the *CytC552* structure and secondary structure distribution along the sequence. α-Helices α1- α5 are colored in red; the heme *c* binding residues Cys, His, and Met are colored in cyan. Residues 23-34, 80-100, and 123-132, presumably involved in the formation of a complex with *TcDH*, are in bold. Loops connecting helixes α1- α5 are marked as L1 – L3. Residues 1-9 are the result of cloning.

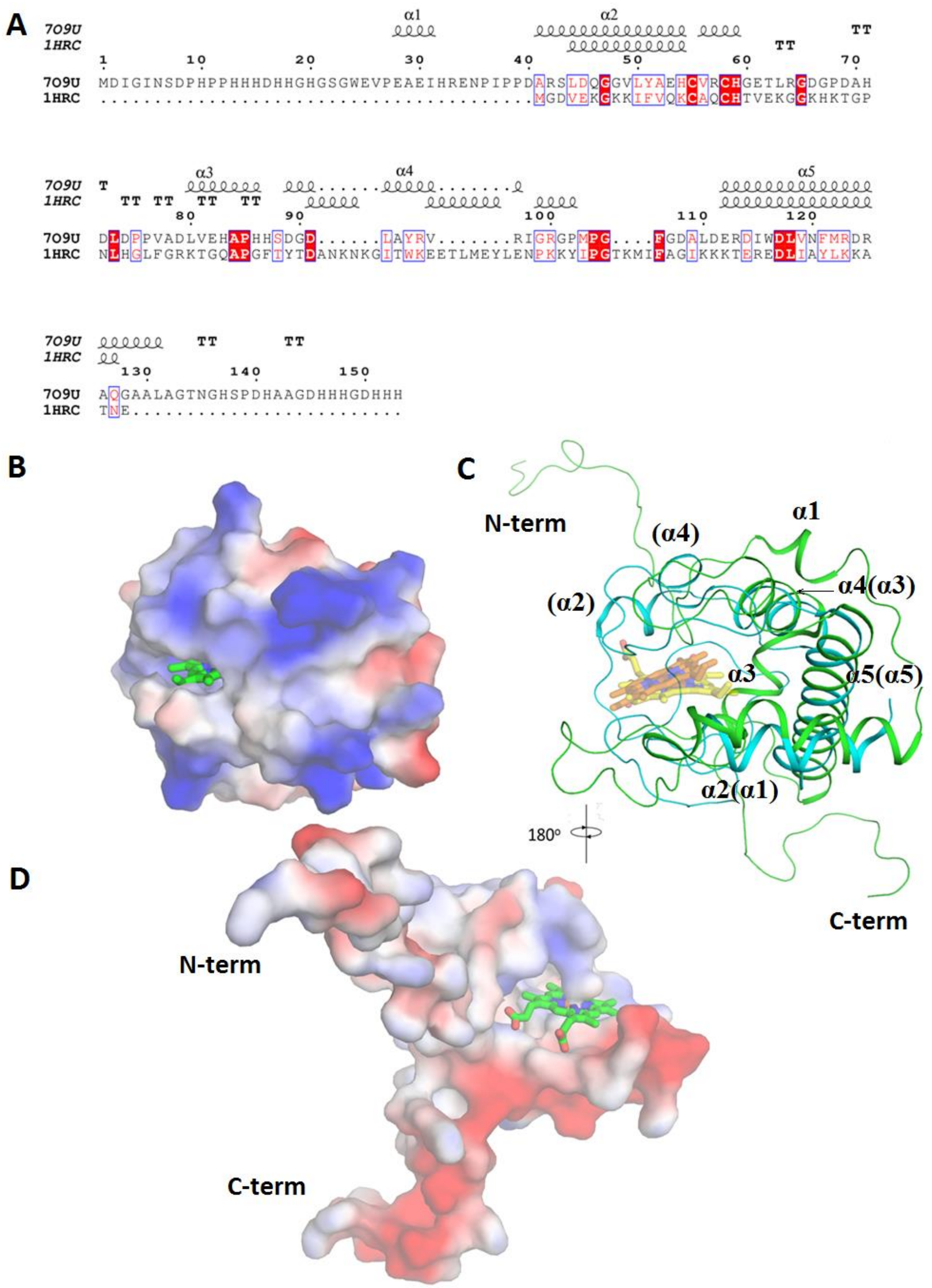

Fig. S5. Structural comparison of *CytC552* (PDB ID 7O9U) and mammalian *hhCytC550* (PDB ID 1HRC) as an example of classical class I cytochromes *c*. **A.** Multiple sequence alignment generated with ESPript (http://espript.ibcp.fr). Highly conserved residues are highlighted in red, semiconserved are colored red. Secondary structure distributions are shown above the aligment**.** The numbering of *CytC552* alpha-helices corresponds to Fig. S4**. B.** Compact alpha-helical globule of *hhCytC550* with the heme *c* group (green) deeply embedded in the core (surface presentation colored by charge). **C.** Superposition of *hhCytC550* (cyan/yellow) and *CytC552* (green/orange) shows that only two longest a-helices have similar positions relatively to the heme *c* group. *HhCytC550* alpha helix numbers are in parentheses. **D.** The N- and C-terminal tails extending from the alpha-helical core and the heme *c* group strongly exposed to the solvent are characteristics of *CytC552*. Surface presentation is colored by charge and heme is colored in green.

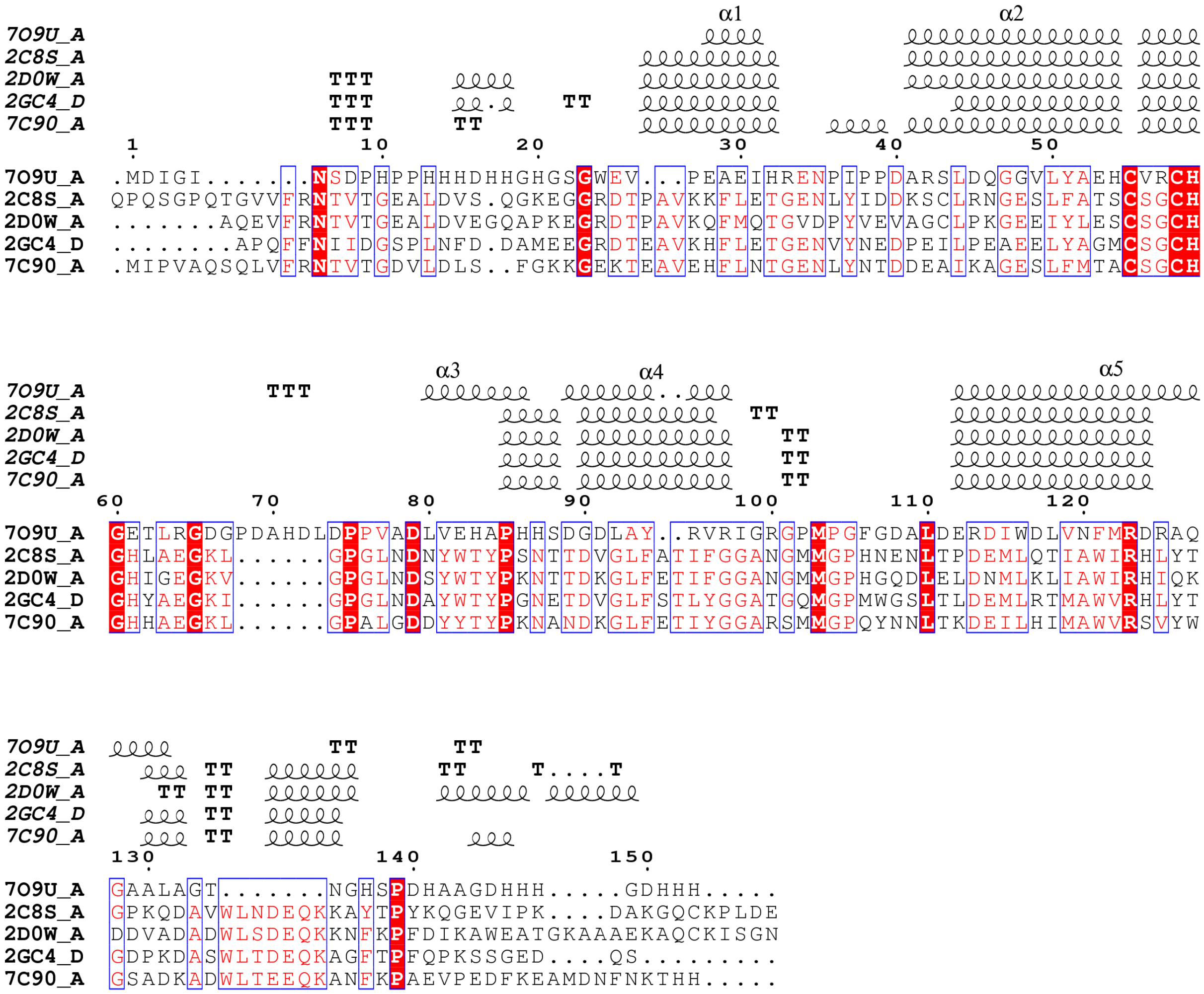

Fig. S6. Multiple sequence alignment of *CytC552* (PDB ID 7O9U chain A) and its closest topological homologs with known 3D structures, which represent large periplasmic cytochromes *c*551/552 (*CytCL*) from the periplasm of methylotrophic and denitrifying bacteria: *Me-CytCL* from *M. extorquens* (PDB ID 2c8s chain A), *Hd-CytCL* from *H. denitrificans* (PDB ID 2d0w chain A), *Pd-CytCL* from *P. denitrificans* (PDB ID 2gc4 chain D) and *Ma-CytCL* rom *M. aminisulfidivorans* (PDB ID 7c90 chain A). Highly conserved residues are highlighted in red, semiconserved are colored red. Secondary structure distributions are shown above the aligment**.** The numbering of the *CytC552* alpha-helices corresponds to Fig. S4**.**

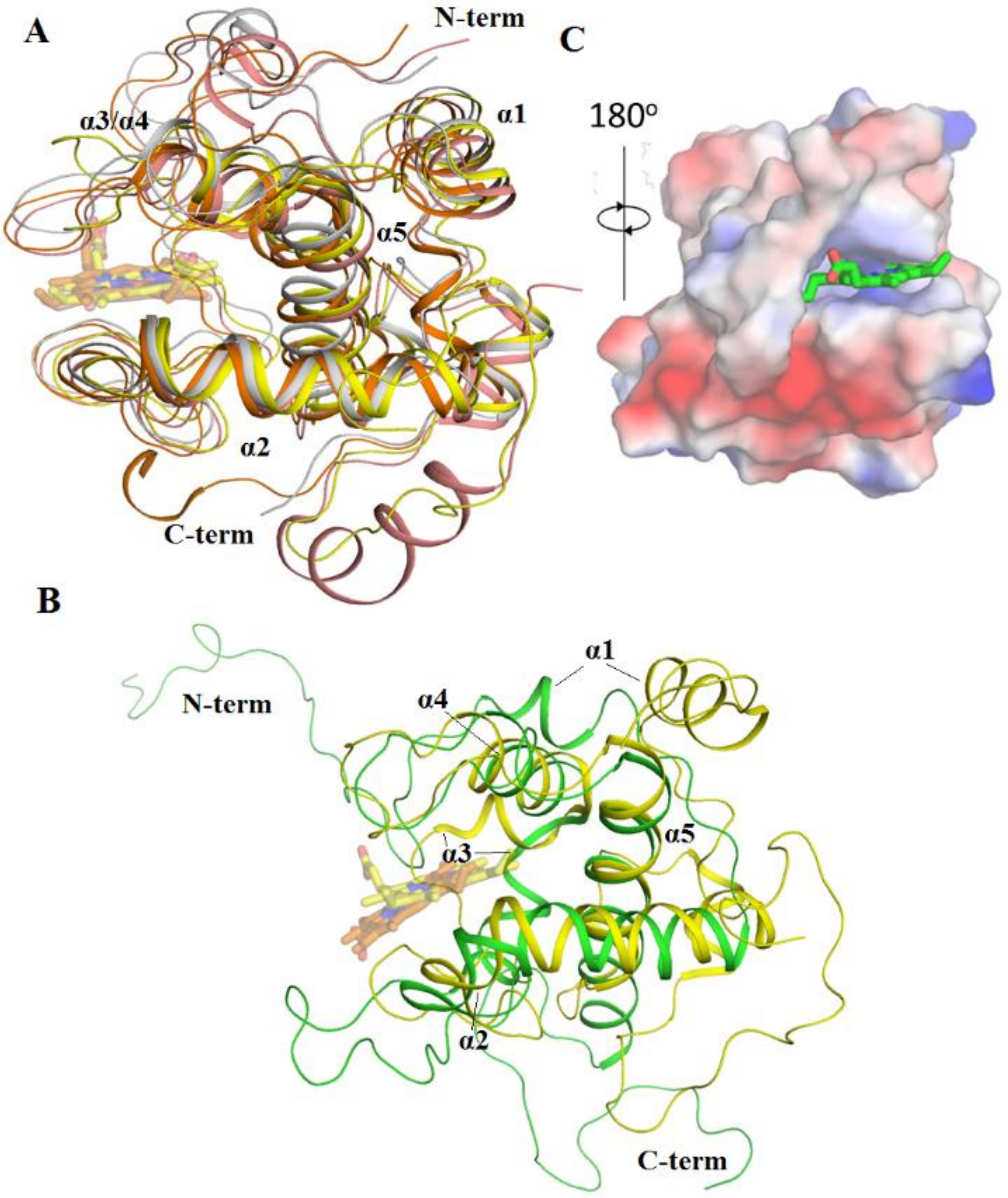

Fig. S7. Similarity and difference of *CytC552* and large periplasmic cytochromes *c*551/552 (*CytCL*) from the periplasm of methylotrophic and denitrifying bacteria. **A.** Superposition of the *CytCL* structures *Me-CytCL* from *M. extorquens* (PDB ID 2c8s, chain A) colored in yellow, *Hd-CytCL* from *H. denitrificans* (PDB ID 2d0w, chain A) colored in salmon, *Pd-CytCL* from *P. denitrificans* (PDB ID 2gc4, chain D) colored in gray and *Ma-CytCL* from *M. aminisulfidivorans* (PDB ID 7c90, chain A) colored in gold. **B.** Superposition of the *CytC552* structure (colored in green/orange) with *Me-CytCL* (colored in yellow). **C.** Partially solvent-exposed heme *c* in the *Me-CytCL* structure (surface presentation colored by charge).

Supplementary methods

Production of recombinant CytC552.

Cloning of the target gene which coded the mature protein without 26 aa signal peptide, was performed according to standard procedures [14]. PCR amplification of the target gene from genomic DNA of *Tv. paradoxus* ARh 1 was done using the primers Cyt F 5' – AAAAGGATCCTCATCCGCCCCACCAC – 3'and Cyt R 5' – AAAAAAGCTTCTAGTGATGGTGATCGCCGTG – 3'. Forward primer contained BamH I restriction site, reverse primer – Hind III restriction site (underlined). Amplification was carried out using Encyclo-polymerase (Evrogen). QIAquick Gel Extraction kit (Qiagen) was used for purification of the PCR product. The resulting DNA fragment was digested with restriction enzymes BamH I and Hind III (Fermentas), and cloned into vector pET-22b(+) (Novagen) pre-treated with corresponding enzymes. The obtained construct was confirmed by DNA sequencing. *Escherichia coli* strain Mach1 (Invitrogen) was used for cloning and strain BL21(DE3) (Novagen), co-transformed with plasmid pEC86 was used for protein production. Cultures were routinely grown in 2x YT medium (16 g/L tryptone, 10 g/L yeast extract, 5 g/L NaCl) or on 2x YT agar plates [14]. Growth medium was supplemented with ampicillin, 100 µg/ml, and chloramphenicol, 34 µg/ml, where appropriate.

The IPTG-induced overexpression of the protein in a rich medium (2xYT) was done [14]. Cells were grown overnight in 2xYT medium with antibiotics at 30°C and 1% of this culture served as inoculum for the fresh media. The cultures were incubated at 30°C and 180 rpm rotation for 8 hours. The protein expression was induced by addition of IPTG up to 0,04 mM, δ-aminolevulinic acid (Sigma) (dALA) up to 0,1 mM and $FeSO_4$ up to 0,1 mM to the cell cultures and they were grown overnight at 30°C and 160 rpm.
